# Pneumococcal colonisation and mucosal immunity in adults with asthma following experimental human challenge

**DOI:** 10.1136/bmjimm-2025-000012

**Published:** 2026-03-19

**Authors:** Jesús Reiné, Seher Raza Zaidi, Simon P Jochems, Sherin Pojar, Elissavet Nikolaou, Elena Mitsi, Esther L German, Caroline M Weight, Angela D Hyder-Wright, Hugh Adler, Helen Hill, Caroline Hales, Victoria Connor, Carla Solórzano, Robert S Heyderman, John D Blakey, David Goldblatt, Stephen B Gordon, Jamie Rylance, Daniela M Ferreira

**Affiliations:** 1 NIHR Oxford Biomedical Research Centre, Oxford Vaccine Group, University of Oxford, Oxford, UK; 2 Department of Clinical Sciences, Liverpool School of Tropical Medicine, Liverpool, UK; 3 Inmunología, Facultad de Medicina, Universidad Complutense de Madrid, Ciudad Universitaria, Madrid, Spain; 4 Center for Infectious Diseases, Leiden University, Leiden University Medical Center, Leiden, Netherlands; 5 Research Department of Infection, Division of Infection and Immunity, University College London, London, UK; 6 Biomedical and Life Sciences, Lancaster University, Lancaster, UK; 7 Liverpool University Hospitals NHS Foundation Trust, Liverpool, UK; 8 Medical School, Curtin University, Perth, Western Australia, Australia; 9 Institute for Child Health, University College London, London, UK; 10 Malawi-Liverpool-Wellcome Trust Clinical Research Programme, Blantyre, Malawi; 11 Institute of Regeneration and Repair, College of Medicine and Veterinary Medicine, University of Edinburgh Division of Clinical and Surgical Sciences, Edinburgh, UK

## Abstract

**Background:**

Individuals with asthma have increased susceptibility to bacterial pneumonia, potentially related to altered mucosal immunity and corticosteroid therapy. Cross-sectional studies suggest higher rates of *Streptococcus pneumoniae* (pneumococcus) carriage in asthma, but the dynamics of colonisation and mucosal immune mechanisms remain unclear.

**Methods:**

We assessed 50 participants with well-controlled asthma (on moderate inhaled corticosteroid therapy) and 151 healthy controls, all experimentally challenged with type 6B pneumococcus. A subset of asthma participants (n=12) who became colonised were rechallenged 6-11 months later with the same pneumococcal isolate. Colonisation rates (from nasal wash), systemic antibody levels and mucosal cellular and cytokine responses were compared between groups.

**Results:**

The colonisation rates were 56% (28/50) in asthma participants and 45% (68/151) in controls (p=0.17). The median duration of colonisation was shorter in asthma participants (14 days, IQR 7–29) compared with controls (29 days, IQR 14–29, p=0.034), although bacterial densities were similar between both groups. Despite an increase in pneumococcus-capsule-specific antibodies following colonisation, 4/12 asthma participants were recolonised after rechallenge. Nasal neutrophil and T cell frequencies, particularly mucosa-associated invariant T cells, were lower in asthma participants compared with healthy controls before challenge. Similarly, 22 out of 30 measured mucosal cytokines were significantly lower at baseline in asthma participants. Despite these deficits, both groups exhibited similar recruitment of monocytes and granulocytes to the nasal mucosa following colonisation.

**Conclusions:**

Adults with asthma demonstrate reduced mucosal immune tone and impaired development of protection against pneumococcal recolonisation, despite preserved innate recruitment and generation of pneumococcal-specific antibodies. These findings identify distinct mucosal immune alterations in asthma that may underlie persistent susceptibility to pneumococcal infection and inform strategies for targeted vaccination and immune modulation.

**Trial registration number:**

ISRCTN16755478.

## Introduction

Asthma is a complex and heterogeneous obstructive respiratory disease characterised by chronic airway inflammation, variable clinical expression and fluctuating airflow limitation. In individuals with asthma, both**l** immune responses and mucosal clearance mechanisms are often impaired, particularly against bacterial pathogens.^1^ This impaired mucosal defence reduces bacterial clearance and increases susceptibility to respiratory infections, thereby predisposing to persistent bacterial colonisation.

*Streptococcus pneumoniae* (pneumococcus) remains the most common cause of bacterial pneumonia worldwide. Large-scale epidemiological studies demonstrate that people with asthma receiving inhaled corticosteroids (ICS) have an increased, dose-dependent risk of pneumonia.^2–4^ Asthma has also been associated with higher rates of nasopharyngeal colonisation by *S. pneumoniae* across both paediatric and adult cohorts,^5–8^ although detailed information on colonisation density and duration is limited. For example, an Italian cross-sectional study reported a 45% pneumococcal colonisation rate in children and adolescents with asthma,^5^ while Finnish army recruits with asthma were twice as likely to be colonised compared with non-asthmatic counterparts.^9^

Mucosal cellular mechanisms are essential for clearance of nasopharyngeal colonisation^10 11^, and impairment of these defences may increase colonisation and the risk of subsequent pneumococcal disease.^1^ Asthma is characterised by airway inflammation, and ICS therapy further modifies mucosal immune responses.^12–14^ Both the disease and its treatment have been linked to reduced frequencies of mucosa-associated invariant T (MAIT) cells in blood and sputum.^12^ Neutrophilic and eosinophilic inflammation also contribute to asthma pathophysiology, particularly in individuals with obesity, who may exhibit reduced responsiveness to Th2-targeted therapies such as anti-IL-5 antibodies.^15^ Given these immune alterations, pneumococcal vaccination is recommended in the UK and USA for individuals with asthma and for those with corticosteroid-induced immunosuppression.^2 5 6^ However, the specific mechanisms underlying susceptibility to pneumococcal colonisation in asthma remain unclear.

In the present study, we used an established human pneumococcal challenge model^16 17^ in people with asthma to elucidate whether the immune responses and protection to pneumococcal colonisation are altered due to the condition itself, or if they are secondary to ICS; and how the altered mucosal immunity presumably mediates the susceptibility to pneumococcal colonisation. To this end, we conducted a longitudinal analysis of the acquisition, duration and density of pneumococcal carriage in people with asthma following pneumococcal challenge and compared that with healthy controls. In addition, we assessed mucosal immune responses to colonisation through collection of minimally-invasive nasal sampling as previously described.^10 18^

## Material and Methods

### Study design

Between June 2016 and April 2018, we recruited 95 participants aged 18–50 years with physician-diagnosed asthma. Medical records were reviewed to confirm history. Participants were at British Thoracic Society (BTS) 2014 treatment steps 2 or 3 (≤800 µg budesonide propionate equivalent; online supplemental Table 1). Based on history and asthma treatment, 50 proceeded to pneumococcal challenge. Full exclusion criteria are in online supplemental Table 2, and recruitment flow is illustrated in online supplemental Figure 1. An overview of the experimental human controlled pneumococcal challenge (EHPC) model is provided in online supplemental Figure 8. A priori exclusions included: post-bronchodilator forced expiratory volume (FEV1) <70%;>1 exacerbation in the prior year; recent use (withi 4 weeks) of antibiotics or oral steroids. None of the participants in either the asthma or healthy control groups had received pneumococcal vaccination prior to enrolment. All participants gave written informed consent, and all the study was conducted in Liverpool (UK).

A structured questionnaire captured demographics and clinical history. Spirometry with reversibility testing was performed per American Thoracic Society (ATS) guidelines (EasyOne, ndd Medical Technologies, Switzerland), along with fractional exhaled nitric oxide (FeNO) (Niox Vero, Circassia, UK), and blood eosinophil count. All participants received a written asthma action plan and peak flow metre (MiniWright, Clement & Clarke, UK).

Inoculation was performed as previously described,^10 17^ administering 80,000 colony-forming units (CFU) of *S. pneumoniae* serotype 6B in 100 µL saline per nostril. This serotype is rarely found in natural colonisation in the UK post-Pneumococcal Conjugate Vaccine (PCV) introduction^16 17 19^ and yields consistent carriage rates across doses (40,000–1 60,000 CFU). Follow-up schedules and sample collections aligned with previous studies^16–18^ (online supplemental Figure 2). At the end of the study, participants who did not clear colonisation before the final two visits received a 3-day course of amoxicillin. A subset of colonised individuals (n=12 randomly selected) was re-challenged 6 months later (online supplemental Figure 2).

We compared results with an age-matched healthy control group (n=151) from a prior experimental human challenge study^20^ from September 2015 to March 2017, which employed identical sampling and methodology. In this study, these participants had no asthma diagnosis and received tetravalent inactivated influenza vaccine either 3 days before or after pneumococcal inoculation.

### Measurement of IgG serum levels against pneumococcus

IgG levels against the 6B pneumococcal capsule were quantified by ELISA, as previously described.^17^ Additionally, 27 pneumococcal protein-specific IgG levels were measured via Meso Scale Discovery (MSD) platform (see online supplemental Methods 1). Serum samples were tested in duplicate at baseline and on day +29 post-challenge. MSD plates were coated with proteins and blocked with 5% BSA. Standards (starting 1:100 dilution of 007sp) were serially diluted, and test sera were diluted 1:100. Quality control sera were run at 1:1000 and 1:2000. Plates were shaken for 1 hour at 700 rpm, washed four times, and detection antibody was added. After a final wash, MSD imaging was performed. Duplicates with >15% coefficient of variation were reassayed.

### Flow cytometry analysis

#### Nasal cell immunophenotyping

Cells were obtained by curettage of the inferior turbinate (Rhino-pro, Arlington Scientific), into PBS with 2.5 mM EDTA and 0.5% Foetal Bovine Serum (FBS) as described.^18^ Samples were stained on ice: first with LIVE/DEAD Fixable Violet Dead Cell Stain (ThermoFisher), followed by an antibody cocktail (online supplemental Methods 2). After washing and filtration through a 70 µm mesh, cells were fixed in 200 µL CellFIX (BD Biosciences) and stored on ice until acquisition. Samples were acquired on a BD LSRII flow cytometer and analysed in FlowJo V.10. Compensation matrices used BD and ThermoFisher beads. Antibody titrations and fluorescence-minus-one controls ensured specificity. Samples with <500 immune (CD45+) or <250 epithelial (EpCam+/CD326+) cells were excluded. Immune cell counts were normalised to epithelial cell numbers.

### Peripheral blood mononuclear cell immunophenotyping

Peripheral blood mononuclear cells (PBMCs) from asthma (n=44) and control (n=13) participants were thawed in prewarmed Roswell Park Memorial Institute medium (RPMI) with 10% FBS, 1% PNS and 50 µg/mL DNase I. Cells were centrifuged (200 g, 10 min), washed, counted and stained at 4°C with LIVE/DEAD stain. Next, cells were stained with CD161-APC, CD4-PerCPCy5.5, CD3-APC-Cy7, CD8-AlexaFluor700, CD45-BV510 and TCRVα7.2-BV786. MAIT cells were gated using a prede-fined strategy (online supplemental Figure 3). Stained PBMCs were washed and analysed as above.

### Mucosal cytokine levels

Nasal lining fluid was collected using nasosorption strips (Hunt Developments), applied to the mucosa for 1 min and stored at –80°C as described.^10 21^ Fluid was eluted with 100 µL diluent buffer and centrifuged (16,000 g, 10 min, 4°C). Cytokines (n=30) were quantified using a 30-plex Luminex magnetic human cytokine kit (ThermoFisher; online supplemental Methods 3) as described.^10^ Samples were analysed in duplicate on an LX200 Luminex. Standards were included in all plates. Undetectable cytokine values were set to the plate-specific detection limit.

All nasal lining fluid samples from participants with asthma and healthy controls were analysed simultaneously using the same lot of the 30-plex Luminex magnetic cytokine kit and the same LX200 Luminex instrument to minimise batch effects. Internal plate controls showed consistent performance across runs; therefore, no additional normalisation to albumin, urea or other house-keeping markers was applied.

### Statistical analysis

Analyses were conducted using SPSS V.22, R V.3.5.0 and Prism V.10.3.1. Area under the curve (AUC) for bacterial density over time was calculated by the trapezoid method on log10 (value+0.01). Colonisation rates were analysed using χ^2^ tests; continuous variables via t-tests or Mann-Whitney U tests. Spearman’s correlation and Wilcoxon tests were used for paired analyses. All tests were two-tailed with significance at p<0.05, adjusting for multiple testing where relevant.

### Sample size

Colonisation was defined by any positive bacterial culture from nasal wash at any point after inoculation. Sample size was based on detecting a 50% relative reduction from a known 49.6% colonisation rate in healthy controls, with 90% power and α=0.05. 52 participants were needed, accounting for 20% potential dropout.

### Patient and public involvement

A patient and public involvement and engagement group composed of healthy volunteers and individuals with asthma who had previously taken part in EHPC studies was actively involved in the development of the study protocol.

## Results

50 participants with asthma were inoculated with *S. pneumoniae* 6B, with no loss to follow-up, and no serious adverse events. None of the participants had been vaccinated with a pneumococcal vaccine. Demographic and clinical baseline parameters from participants are shown in online supplemental Table 3 and online supplemental Table 4. Median age in people with asthma and control groups was 22 years (IQR 20–26) and 20 years (IQR 19–22), with 38 (76%) and 82 (54%) female, respectively.

Participants with asthma had well-controlled asthma: the median baseline blood eosinophil count was 0.2×10^9^/L (IQR 0.1–0.4); the median FEV1% predicted was 95% (91–100); the median FeNO was 25.5ppb (13.8–49.2); and the median asthma control score (ACT) was 22 (20–24) (online supplemental Table 4). Those on step 2 and step 3 treatment had similar blood eosinophil counts, FeNO and ACT (online supplemental Table 4). We confirmed an expected positive correlation between FeNO and blood eosinophils (p<0.0001, r=0.57).

### Factors associated with establishment of pneumococcal colonisation in people with asthma

Pneumococcal 6B inoculation doses are shown in online supplemental Table 3 for each group: the median dose per nostril was 83,417 CFU (78,333–85,292) and 78,834 CFU (IQR 76,333–84,167) for individuals with asthma and healthy controls, respectively.

In the asthma cohort, participants who became colonised (positive bacterial culture from nasal wash at any timepoint) following challenge and participants who did not become colonised had similar ACT baseline characteristics (online supplemental Table 5). FeNO levels were not significantly different at baseline (colonised participants median 20.5 ppb (IQR 13.5–37.5) compared with non-colonised participants (29.5 (IQR 13.5–54.75), p=0.287). One participant was colonised at baseline with *S. pneumoniae* serotype 9 and subsequently became experimentally colonised with 6B, with both serotypes identified at one time point.

**Figure 1:**
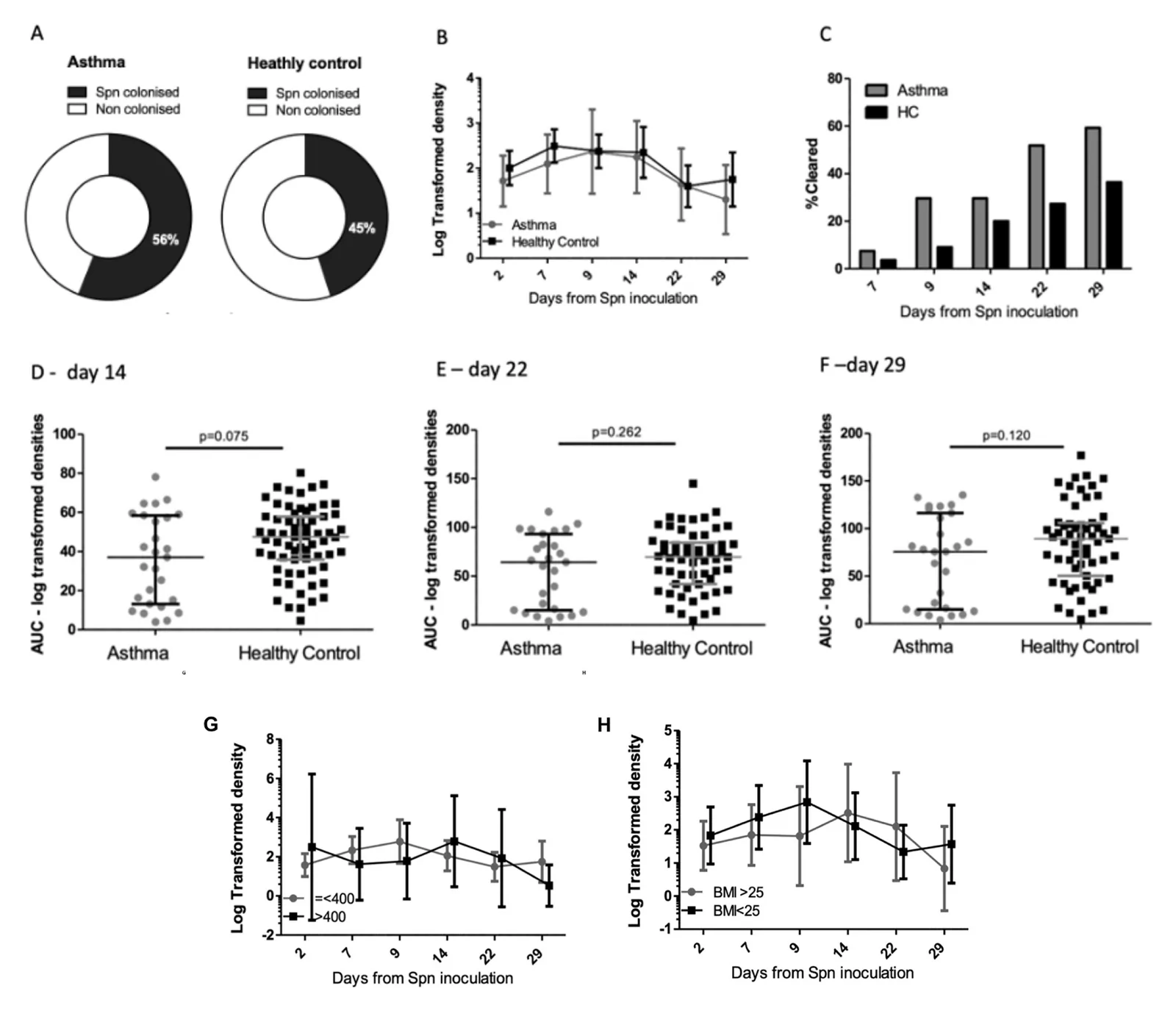
Pneumococcal acquisition and colonisation in asthma and healthy controls (HC). (A) Percentage of participants colonised at any time post-inoculation. (B) Bacterial colonisation density at each time point for all positive participants (asthma: n=19, 22, 17, 19, 12, 11 at days 2, 7, 9, 14, 22, 29, respectively; HCs: n=61, 62, 60, 50, 43, 35 at days 2, 7, 9, 14, 22, 29). (C) Cumulative clearance of colonisation, defined as the first time point with a negative nasal wash after an earlier positive result, comparing asthma and HCs at each time point. Clearance rates at each time point for asthma and healthy controls. (D–F) Area under the log-transformed bacterial colonisation density curve for participants who were colonised with pneumococcus at any time and attended all visits up to: (D) Day 14 (asthma: n=27, HCs: n=67), (E) Day 22 (asthma: n=27, HCs: 57), (F) Day 29 (asthma: n=27, HCs n=59). Median and IQR are shown, with p values from Mann-Whitney U test. (G) Bacterial colonisation density in asthma participants according to inhaled corticosteroids (ICS) dosage (ICS; >400 µg vs ≤400 µg) all among colonised participants. (H) Stratification of asthma participants by body mass index (BMI; >25 kg/m^2^ vs <25 kg/m^2^) all among colonised participants. Values are log-transformed, with bars representing the mean and 95% confidence interval. AUC, area under the curve.

The body mass index (BMI) was higher in colonised than in non-colonised participants with asthma (median 24.7 kg/m^2^ (IQR 24.1–29.0) and 23.5 kg/m^2^ (20.1–26.4) p=0.019), respectively. However, there was no association between colonisation versus BMI and/or ICS treatment step (figure 1).

### Experimental pneumococcal colonisation in people with asthma

Experimental induced pneumococcal colonisation rates were not significantly different in people with asthma compared with healthy controls (28/50 (56%) vs 68/151 (45%), respectively, p=0.178) (figure 1A, online supplemental Table 6). Pneumococcal density at all time points was similar among colonised individuals of the two groups (figure 1B, online supplemental Table 6).

Despite a similar pneumococcal acquisition rate and density between groups, we observed a reduced duration of colonisation in people with asthma (14 days, IQR 7–29) compared with healthy controls (29 days, IQR 14–29, p=0.034, Mann-Whitney U test) (figure 1C, online supplemental table 6). AUC of colonisation density over time (days 14, 22 and 29) was similar between people with asthma and healthy controls (Figure 1D–F).

We then assessed whether either ICS doses or BMI was associated with colonisation density. The density at any timepoint and overall, as calculated by AUC, was comparable in people with asthma regardless of ICS dose (>400 µg BDP) or BMI >25 kg/m^2^) (Figure 1G,H).

### Experimental homologous pneumococcal rechallenge in people with asthma

Previous reports demonstrated complete protection in ten healthy individuals against pneumococcal recolonisation with the same serotype 6B up to 1 year after initial experimental colonisation.^17^ To assess whether colonisation induces similar protection in people with asthma, 12 colonised individuals from the asthma group were rechallenged with the same strain 6–11 months after the first inoculation. Of these, four became recolonised (33.3%) representing a non-significant difference in colonisation rate from the 56% in the primary challenge (relative risk 59.5%, 95% CI 25.8% to 138%, Fisher exact p=0.20). The time between first colonisation episode and rechallenge was not different in those who became recolonised compared with those who did not (251 days (IQR 237–292) vs 238 days (IQR 223–300)), respectively.

**Figure 2:**
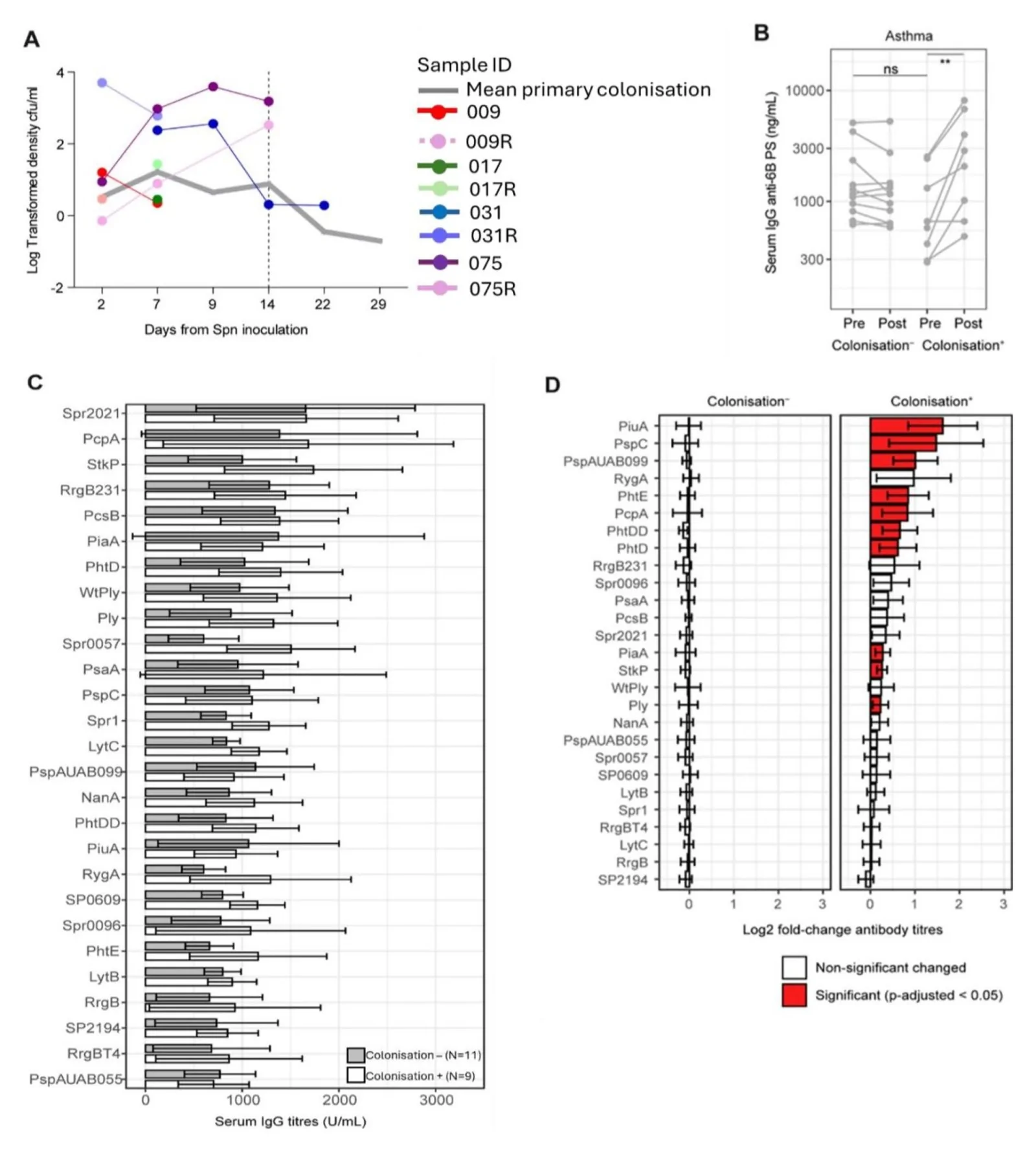
Immunising effect of colonisation in people with asthma. (A) Bacterial colonisation density during challenge and rechallenge. Log-transformed densities at each time point are shown. The median log-transformed colonisation density during the primary episode for all asthma participants is indicated by a grey line (from figure 1D). Densities are shown for individuals who were positive upon re-challenge, with rechallenge samples indicated by ‘R’ after their ID. Sampling was limited to day 14, as indicated by a dashed line. (B) Anti-6B polysaccharide (PS) IgG titres in serum of asthmatic individuals before and after challenge, stratified by colonisation status. **p=0.004 by paired t-test. (C) Baseline IgG titres against 27 pneumococcal proteins, measured by multiplex assay (MSD). Mean and 95% CIs are shown for colonised (grey) and non-colonised (white) participants. (D) Anti-protein IgG titres as log2 fold-change from baseline, stratified by colonisation status. Mean and 95% CIs are shown. Red bars indicate proteins with significant changes in titres following colonisation (p<0.05 by paired t-test, adjusted for multiple testing using Benjamini-Hochberg). MSD, Meso Scale Discovery.

Taken into consideration the limitation of our sample size, the density observed during the second colonisation episode did not appear substantially different from the first episode (figure 2A). Of these four individuals, three cleared colonisation within 14 days of rechallenge.

### Pneumococcal IgG antibody levels before and after colonisation

To understand whether colonisation was an immunising event in people with asthma and why it did not lead to complete protection from rechallenge, as previously reported in healthy adults, we investigated anti-CPS and anti-protein antibody responses in serum samples before (baseline) and after (day+29) challenge (figure 2B). At baseline, levels of serum CPS IgG were not significantly different between individuals who became colonised (colonisation+) or not (colonisation–). We observed an increase in levels of CPS IgG following colonisation (day+29) compared with baseline in colonisation+ individuals only (median three-fold increase, p=0.0039 paired t-test). This was comparable to our previous observations in healthy adults of median 2.6-fold increase at day +21 compared with baseline.^22^

We also evaluated serum IgG levels against 27 pneumococcal proteins before and after colonisation in individuals with asthma. At baseline, no significant differences in serum IgG levels were observed between colonised (colonisation+) and non-colonised (colonisation−) participants with asthma for any of the 27 pneumococcal proteins (figure 2C). However, we observed a significant increase in IgG levels against 10 pneumococcal proteins (PspC, PspAUAB099, PhtE, PcpA, PhtDD, PhtD, PiaA, StkP and Ply) in the colonisation+ group following challenge (figure 2D).

Collectively, these antibody findings in people with asthma are similar to what has been previously described in healthy individuals, where levels of antibody against pneumococcal proteins and capsule polysaccharide (PS) increase following 6B experimental induced pneumococcal colonisation, but their baseline levels do not predict acquisition risk.^17 22–24^

### Nasal immune cell populations in individuals with asthma compared with healthy controls before colonisation

To assess whether differences in nasal immune populations were underlying the altered dynamics of pneumococcal colonisation in individuals with asthma, we immunophenotyped nasal immune cells using minimally-invasive nasal curettes by flow cytometry (online supplemental Figure 4) as previously described.^10 18^ We assessed both absolute immune cell numbers and immune cell numbers normalised to observed epithelial cell counts to correct for variable curettage yield.

**Figure 3:**
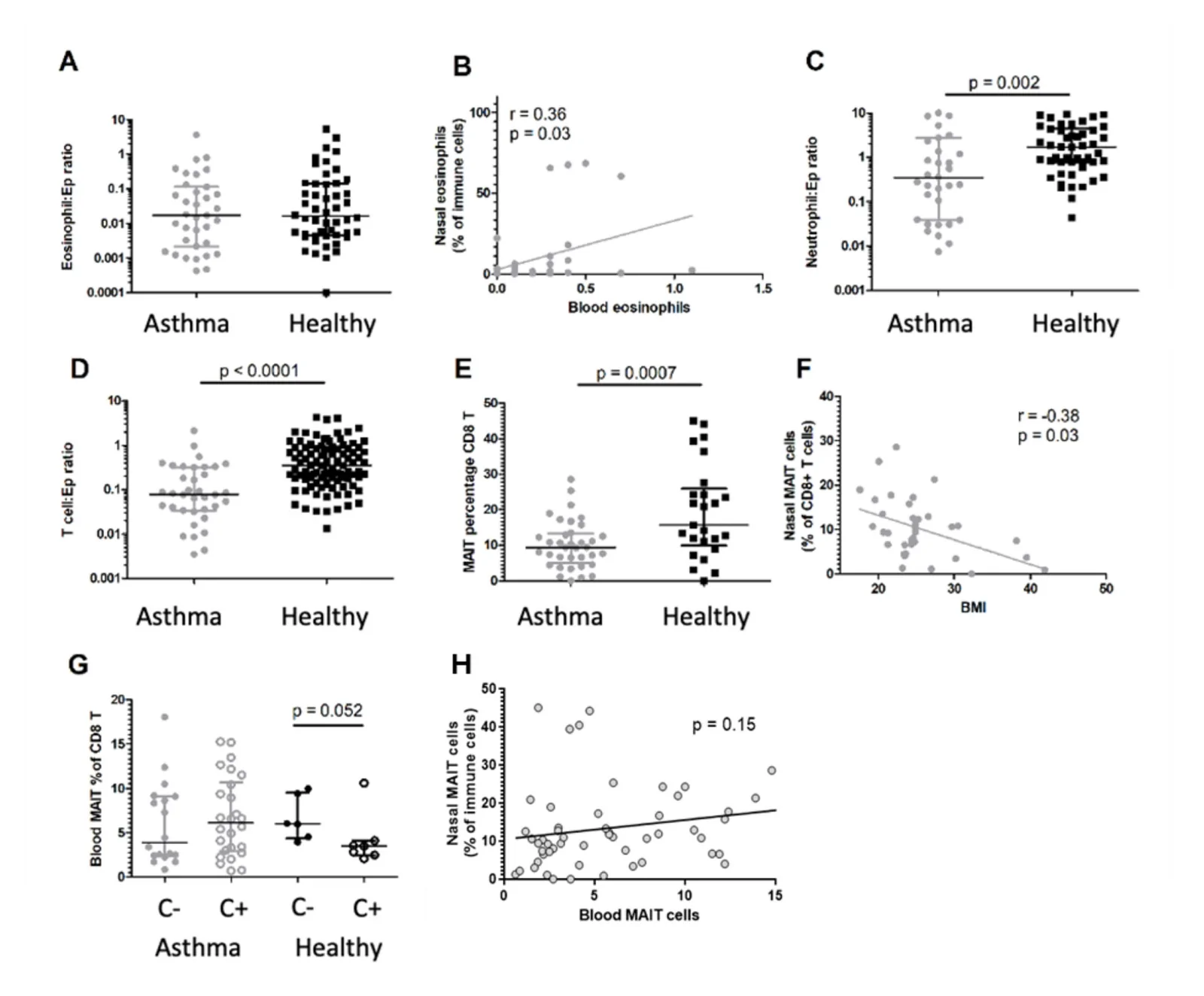
Nasal immune profiles in asthma and healthy control at baseline. (A) Eosinophil levels normalised to epithelial cells in individuals with asthma (closed grey circles) and healthy control (closed black squares). Symbols represent individual participants, with group median and IQR overlaid. (B) Correlation between blood eosinophil count (×1000/µL) and nasal eosinophil levels, with linear regression (line) and Spearman correlation (r and p values) shown. (C) Neutrophil levels normalised to epithelial cells in people with asthma and healthy control participants. (D) CD3+ T cell levels normalised to epithelial cells in asthma and healthy control participants. (E) Percentage of mucosal-associated invariant T (MAIT) cells among nasal CD8+ T cells, with median and IQR overlaid and individual symbols shown. (F) Linear correlation between body mass index and MAIT cell frequency (Spearman rank test). (G) MAIT cell levels among blood CD8+ T cells at baseline in asthma and healthy control participants, stratified by colonisation status (colonised C+ and non-colonised C−) Mann-Whitney U tests were used to compare colonisation status due to non-normal distribution of data. (H) Correlation between peripheral blood MAIT cell levels and nasal MAIT cell levels, with linear regresion (line) and Spearman correlation (p value) shown.

Nasal eosinophil frequency was similar in people with well-controlled asthma and healthy controls (figure 3A), with only a modest positive correlation with circulating blood eosinophil levels in asthma patients (r=0.36, p=0.03, figure 3B). This supports the conclusion that asthma was well-controlled in these participants, consistent with our observations of FeNO levels and eosinophil counts.

Nasal neutrophil frequency was significantly lower in individuals with asthma on ICS compared with healthy individuals (median neutrophil/epithelial cell ratio 0.35 (0.038–2.75) vs 1.72 (IQR 0.75–4.43), p=0.002,figure 3C). A similar pattern was observed in nasal T cells (median T/epithelial cell ratio 0.079 (0.034–0.32) and 0.36 (0.17–0.89) in people with asthma compared with healthy controls respectively, p<0.0001, figure 3D).

MAIT cells were notably reduced within the T cell population in people with asthma (median proportion of total CD8^+^ T cells 9.4% (IQR 5.0–13.3)) compared with healthy individuals (median 15.8% (IQR 9.9–25.9), p=0.0007, figure 3E). Nasal MAIT levels were negatively correlated with BMI in people with asthma (r=−0.38, p=0.03, figure 3F). As systemic MAIT cells were previously associated in protection against pneumococcal colonisation,^25^ peripheral blood MAIT cells were characterised at baseline by flow cytometry (online supplemental Figure 4). However, their frequency did not differ between people with asthma (n=44) compared with healthy individuals (n=13)(figure 3G). Within the healthy population cohort, MAIT cell frequency was slightly higher than in those protected from colonisation (median 6.0% vs 3.5%, p=0.052 within CD8^+^ T cells) but did not differ regardless of their colonisation status in people with asthma.

In addition, no positive correlation was observed between MAIT cells from peripheral blood compared with nasal MAIT cells in people with asthma. MAIT cell frequencies were notably lower in peripheral blood compared with nasal mucosa (figure 3H).

In summary, significant differences in nasal mucosa immune cell populations were observed between people with asthma and healthy individuals before colonisation, with a reduced neutrophil, T cell and notably MAIT cell frequencies within the asthma patients, presumably due to therapy.

### Nasal cell population frequencies in asthma individuals following pneumococcal challenge

To delineate the impact of pneumococcal colonisation on mucosal immune cell populations in individuals with asthma, and to link immune recruitment to colonisation dynamics, we conducted a longitudinal analysis of nasal immune cell populations by flow cytometry following colonisation (days 2, 7, 9 and 29).

**Figure 4:**
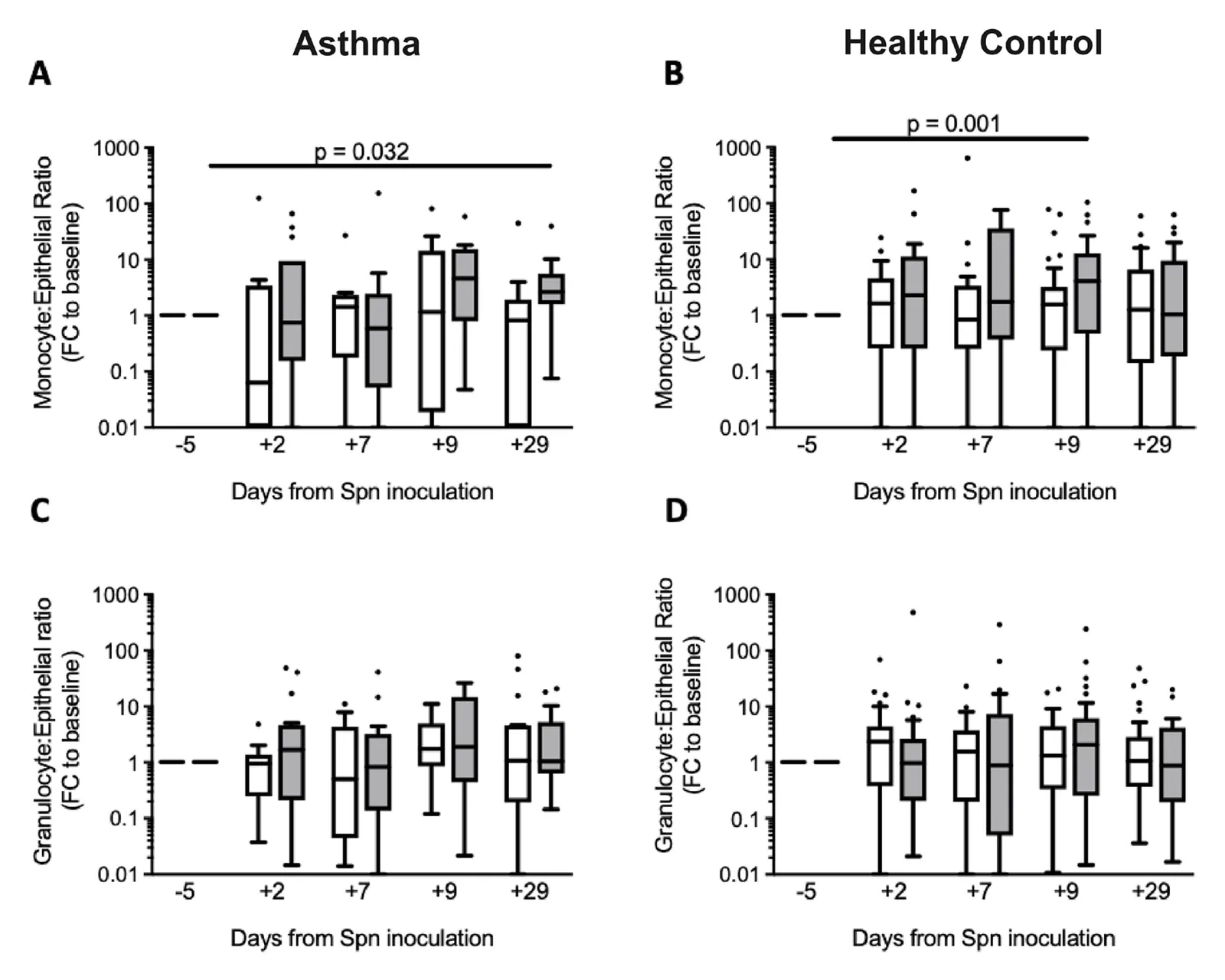
Longitudinal measurements of monocytes and granulocytes in nasal mucosa following pneumococcal challenge. Baseline-normalised monocyte levels in (A) asthma and (B) healthy control and granulocyte levels in (C) asthma and (D) healthy controls are shown. Cell counts were normalised to epithelial cells. Carriage positive is indicated with grey boxplots, and carriage negative is indicated with white boxplots in each case. Turkey boxplots are presented, with results from paired-Wilcoxon rank tests comparing changes from baseline (−5) to days 2, 7, 9 and 29. FC, fold change; Spn, *S. pneumoniae*.

We observed an increase in monocytes, when normalised by total epithelial numbers, for both the asthma cohort at day+29 (p=0.032) and healthy controls at day+9 (p=0.001) when compared with baseline (figure 4A,B). Looking at absolute immune cell numbers, in the cohort of people with asthma, we observed a positive trend towards the recruitment of monocytes at day +9 post-colonisation compared with baseline (median fold change (FC)=3.84, (IQR 1.15–9.38), p=0.055). We confirmed this observation in health controls (median FC=3.63, (IQR 1.24–11.43), p=0.002, online supplemental Figure 5A,B), consistent with our previous findings.^10^ Furthermore, the nasal monocyte population remained increased at day +29 in people with asthma (median FC=2.23 (IQR 1.50–4.25), p=0.037) and healthy controls (median FC=1.72 (IQR 0.35–11.43), p=0.049, see online supplemental Figure 5A,B).

Granulocytes showed a non-significant increase, after normalisation to the number of epithelial cells, in people with asthma (median FC=1.90) and healthy controls (median FC=2.07) at day+9, (figure 4C,D). Absolute numbers of nasal granulocytes were significantly increased in colonised people with asthma at day+9 (median FC=1.78, (IQR 1.09–8.06), p=0.044), but were no longer increased at day+29 (median FC=1.84, (IQR 0.88–5.04), p=0.17, online supplemental Figure 5C). In healthy controls, numbers of granulocytes were increased at day+7 (median FC=3.17, (IQR 1.05–7.37), p=0.009), returning to baseline levels at day+29 (median FC=1.03, (IQR 0.41–3.05), p=0.89, online supplemental Figure 5D).

When examining granulocytes separately, neither neutrophils (defined as CD16^+^ granulocytes) nor eosinophils (defined as CD16^−^ granulocytes) were significantly recruited. Again at day+9, there were non-significant increases in neutrophils (median FC=1.6) and eosinophils (median FC=2.0) in people with asthma (online supplemental Figure 6).

No differences were observed in classical T cell populations, CD4^+^ and CD8^+^ T cells, following colonisation in people with asthma (online supplemental Figure 7A,B). Additionally, other immune cell populations, such as mast cells, which play a crucial role in asthma pathogenesis by interacting with other immune cells and amplifying the inflammatory response, showed no changes over time between baseline and post-colonisation (online supplemental Figure 7C).

### Nasal cytokine levels in asthma individuals following pneumococcal colonisation

To assess the inflammatory responses in asthma individuals before and after pneumococcal colonisation, we analysed nasal lining fluid for 30 cytokines and immune proteins using multiplex detection by Luminex at base-line, and following pneumococcal colonisation (days 2, 7, 9 and 29).

**Figure 5:**
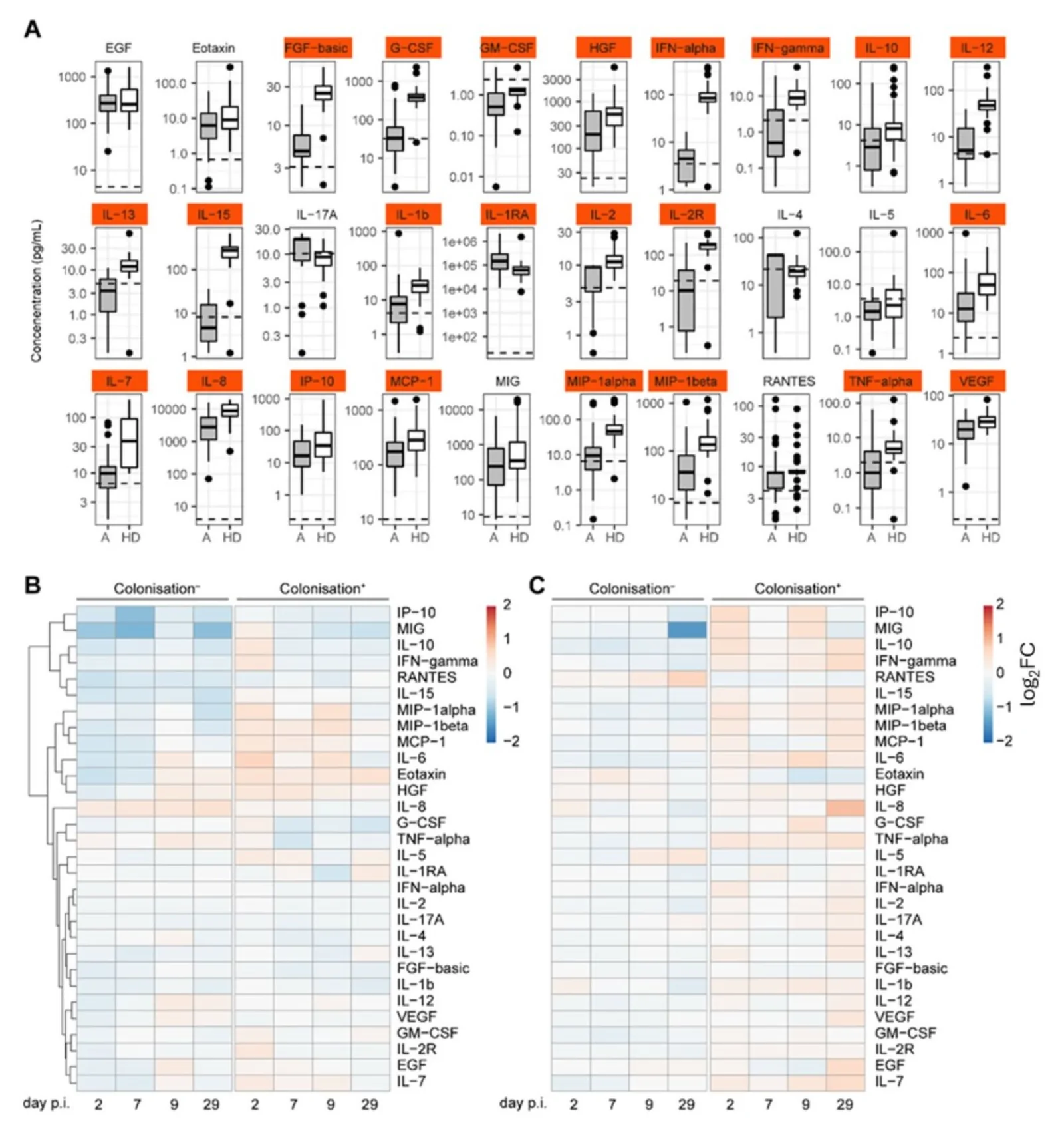
Nasal lining fluid cytokine analysis. (A) Baseline levels of 30 cytokines in individuals with asthma (A, grey) and healthy controls (H, white). Cytokines that were significantly different are highlighted in red, with non-significant cytokines in white. Analysis was performed using t-tests on log-transformed values, followed by Benjamini-Hochberg correction for multiple testing (p=0.01 threshold). Boxplots represent data for each group, with dashed horizontal lines indicating the average value of the lowest standard across all plates for each cytokine. (B) Heatmap of cytokine levels over time in people with asthma (n=55, 26 colonised) following inoculation. (C) Heatmap of cytokine levels over time in healthy controls (n=63, 30 colonised) following inoculation. The mean log2 fold-change compared to baseline is shown per day and cytokine, stratified by colonisation status (colonised C+ and non-colonised C–). G-CSF, Granulocyte Colony-Stimulating Factor; FGF, Fibroblast Growth Factor; HGF, Hepatocyte Growth Factor.

Remarkably, consistent with lower immune cell counts and effective control of asthma in this cohort, baseline analysis revealed a reduced concentration of most of the cytokines analysed (22/30) in individuals with asthma compared with healthy controls (figure 5A; online supplemental table 7). The anti-inflammatory cytokine IL-1RA was elevated in individuals with asthma, while Th-2 cytokines IL-4 and IL-5 remained unchanged at baseline (figure 5A). Baseline nasal cytokine levels did not differ between colonised and non-colonised participants within either the asthma cohort or the healthy donor cohort (online supplemental Figure 12). Following pneumococcal colonisation, no differences in nasal cytokine levels were observed between people with asthma and healthy individuals, regardless of colonisation status. No significant changes were observed by paired t-test with Benjamini-Hochberg correction for multiple testing (figure 5B,C; online supplemental table 8). This absence of inflammation is consistent with our previous observations, which showed reduced immune cell frequencies in the asthma group.

## Discussion

Asthma encompasses a spectrum of chronic inflammatory airway disorders with variable clinical expression and immune dysregulation. These immune alterations can heighten susceptibility to respiratory infections and bacterial colonisation, which in turn may influence disease control and exacerbation risk. Using the (EHPC) model we investigated, under controlled conditions, pneumococcal colonisation acquisition and control and mucosal immunity in adults with asthma receiving ICS and compared with those of healthy age-matched controls.

Colonisation rates and bacterial density in asthma participants did not differ significantly from healthy controls, contrary to previous cross-sectional studies reporting increased colonisation in asthma.^5^ To further explore this, we stratified asthma participants by ICS dose and BMI, but neither factor significantly affected colonisation density over time.

Comparing with earlier studies proved difficult due to methodological heterogeneity, many previous cohorts included patients with varying asthma severity, incomplete treatment documentation and self-reported diagnoses. Such variation may reflect populations with more severe or uncontrolled disease and potentially greater susceptibility to colonisation due to microbiota changes or immune dysregulation. In contrast, our well-characterised and controlled cohort likely had a more stable mucosal immune environment.

In paediatric populations, pneumococcal colonisation is strongly associated with asthma exacerbations,^26^ yet in our adult cohort, no significant airway inflammation was observed following colonisation. Biomarkers such as FeNO and nasal cytokines remained unchanged following challenge/colonisation. This might reflect ICS-induced suppression of type-2 inflammation,^27^ the possibility that we missed early immune responses (before 48 hours),^28^ or that colonisation is a secondary consequence rather than a cause of asthma exacerbations.

Unexpectedly, asthma participants cleared colonisation faster than healthy controls, despite reduced nasal immune cell frequencies and cytokine levels at baseline. This finding appears paradoxical but may reflect differences in mucosal immunity associated with ICS treatment or host–pathogen interactions specific to this population.

Regarding the immunising effect, colonisation led to a three-fold increase in serum IgG to 6B capsular polysaccharide, in line with previous findings.^17^ Additionally, antibodies to 10 of the 27 pneumococcal proteins tested increased post-colonisation. However, neither base-line antibody levels nor the magnitude of this response correlated with protection on rechallenge, indicating that humoral responses alone may not mediate protection. Given the limited sample size of the rechallenge arm, these findings should be considered exploratory and hypothesis-generating, and any apparent differences in protection against homologous recolonisation between adults with asthma and healthy controls will need to be formally evaluated in larger, prospectively designed studies.

Baseline nasal neutrophils and T cells, including MAIT cells, were significantly reduced in asthma participants, regardless of ICS dose. As all participants with asthma were receiving ICS therapy, we cannot fully disentangle disease-related from treatment-related effects, and the observed mucosal immune alterations likely reflect the combined impact of asthma and its therapy. However, stratified analyses by treatment intensity did not demonstrate an association between corticosteroid exposure and baseline nasal immune cellularity or cytokine profiles, suggesting that the reduced mucosal immune tone observed is unlikely to be explained solely by BTS treatment (online supplemental figures 9-11). Prior studies reported MAIT cell depletion in blood and sputum in asthma,^12^ but here the reduction was limited to the nasal mucosa, with no differences in circulating MAIT cell levels. This suggests compartmentalised immune suppression, potentially driven by ICS or local factors.

Lower cytokine levels in nasal lining fluid of asthma participants further support the notion of mucosal immune suppression. This is consistent with their high ACT scores, indicating well-controlled disease. Despite these baseline differences, the dynamics of immune cell recruitment after colonisation were similar between groups, particularly monocyte and granulocyte recruitment, previously reported in healthy individuals,^10^ suggesting preserved innate responses in well-controlled asthma.

Interestingly, elevated BMI was associated with increased risk of experimental colonisation in asthma participants, although other clinical markers (FeNO, eosinophils, spirometry) were not. This aligns with known associations between blood eosinophils and FeNO,^29^ and a positive correlation between nasal and blood eosinophil counts. While obesity is a known treatable trait in asthma,^30^ its role in colonisation remains unclear, particularly since we lacked BMI data for the control group. Our cohort’s low use of systemic corticosteroids reduces the likelihood that obesity resulted from treatment effects.

Several limitations should be acknowledged. The sample size was modest and had a 3:1 female-to-male ratio, limiting generalisability.^31^ Recruitment was constrained by safety concerns and strict criteria, excluding those with severe asthma and precluding exploration of endo-types potentially more susceptible to colonisation. Our focus on well-controlled asthma, with few exacerbations and mild disease, ensured participant safety but may not represent the broader asthma population. In addition, antibody levels were not assessed in the nasal mucosal compartment, as nasal wash samples were optimised for microbiological assessment of pneumococcal colonisation; antibody measurements in this study were therefore restricted to systemic responses measured in serum.

Microbiota alterations, potentially driven by ICS or antibiotics, could also affect colonisation duration. We minimised confounding by excluding recent antibiotic users and selecting stable asthma patients. Still, future studies should explore the microbiome, colonisation relationship in more diverse asthma phenotypes, including those with poorly controlled or severe disease.

This first controlled human infection study in adults with asthma shows that, although pneumococcal acquisition rates were similar to healthy individuals, colonisation was shorter and mucosal immune responses were attenuated. Elevated BMI increased susceptibility, while antibody responses alone did not provide protection. These findings highlight distinct mucosal immune alterations associated with ICS use and underscore the need to investigate how asthma severity influences susceptibility to pneumococcal colonisation and infection.

## Supplementary Material

This content has been supplied by the author(s). It has not been vetted by BMJ Publishing Group Limited (BMJ) and may not have been peer-reviewed. Any opinions or recommendations discussed are solely those of the author(s) and are not endorsed by BMJ. BMJ disclaims all liability and responsibility arising from any reliance placed on the content. Where the content includes any translated material, BMJ does not warrant the accuracy and reliability of the translations (including but not limited to local regulations, clinical guidelines, terminology, drug names and drug dosages), and is not responsible for any error and/or omissions arising from translation and adaptation or otherwise.

Supplementary Material

## Data Availability

Data are available on reasonable request.

## What Is Already Known on This Topic

⇒ People with asthma are at increased risk of bacterial pneumonia, a susceptibility partly attributed to inhaled corticosteroid (ICS) use and impaired mucosal immunity. Cross-sectional studies have suggested higher rates of *Streptococcus pneumoniae* colonisation in asthma, but the cellular and molecular mechanisms driving this remain poorly defined.

### What This Study ADDS

⇒ This is the first controlled human infection study to investigate pneumococcal colonisation and mu-cosal immune responses in adults with asthma. Adults with well-controlled asthma showed similar acquisition rates but shorter colonisation duration than healthy controls and showed limited protection against homologous nasal recolonisation following primary inoculation. Baseline nasal immune profiles, particularly neutrophils, T cells, mucosa-associated invariant T cells and cytokine levels, were significantly reduced, yet innate immune recruitment, as well as induction of specific antibodies, following colonisation was preserved.

### How This Study Might Affect Research, Practice Or Policy

⇒ These findings reveal that well-controlled asthma is associated with altered mucosal immunity and limited immunological memory following pneumococcal exposure. Understanding these mechanisms could inform vaccine design and prevention strategies in asthma and other corticosteroid-treated populations.
