# Supplementary material for: Pneumococcal colonisation and mucosal immunity in adults with asthma following experimental human challenge
Source: BMJ Immunol. Author manuscript; Available in PMC 2026 Nov 1. (PMC7619667; doi:10.1136/bmjimm-2025-000012)
Supplement: Supplementary file 1 [file EMS219694-supplement-Supplementary_Material.pdf]

Supplemental material: Tables

Supplemental Table 1: British Thoracic Society (BTS) treatment steps according to the 2014 asthma management guidelines (Thorax 2014; 69 Suppl 1: 1–192; PMID: 25323740). The shaded area indicates the cohort of patients recruited for the present study.

BTS treatment steps - Asthma population cohort

1	**Mild intermittent asthma**	Inhaled Short Acting β2 Agonist (SABA) as required
2	**Regular preventer therapy**	SABA+
		Inhaled corticosteroids (ICS) 200-400µg
3	**Initial add-on therapy**	SABA+
		Add an inhaled long acting β2 agonist (LABA)
		Increase ICS up to 800µg
		Consider other therapies - Leukotriene Receptor Antagonists (LRA) and slow-release theophylline
4	**Persistent poor-control**	SABA+
		Increase ICS up to 2000µg
5	**Continuous or frequent use of oral steroids**	SABA+LABA+2000µg ICS
		Regular ICS to control symptoms

Supplemental Table 2: Exclusion Criteria for EHPC Asthma

EHPC exclusion criteria in people with asthma	
1	Close physical contact with at-risk individuals (children under 5yrs, immunosuppressed adults)
2	History of drug or alcohol abuse - drinking over the recommended alcohol intake limit (more than 14 units of alcohol per week)
3	Current Smokers or ex-smoker with a significant smoking history (>10 pack years)
4	Taking medications that may affect the immune system e.g. oral steroids, steroid nasal spray, antibiotics, and disease-modifying anti-rheumatoid drugs
5	Antibiotic use in the past 4 weeks
6	Any acute illness – unexplained symptoms in the preceding 14 days
7	Having received any antibiotics, oral steroids or nasal steroid spray in the preceding 28 days
8	More than one asthma exacerbation (as defined by ATS) in the last twelve months
9	Taking anticoagulants
10	History of culture-proven pneumococcal disease
11	Allergy to penicillin/amoxicillin
12	Simultaneously involved in another clinical trial unless observational or in follow-up (non-interventional) phase.
13	History of significant cardiorespiratory disease (excluding stable hypertension, and asthma at treatment step 2 and 3), or diseases associated with altered immunity, including diabetes, alcohol abuse, malignancy, rheumatological conditions
14	Pregnancy
15	Taking any medications except those on the “allowed list” (statins, anti-hypertensives, antidepressants, bisphosphonates, hormone replacement therapy, vitamin supplements, anti-acid medications, nicotine replacement therapy (NRT), inhaled corticosteroids up to 800µg BDP equivalent, inhaled β2 agonists, leukotriene receptor antagonist
16	Participants with a history of pneumococcal vaccination were excluded

Reiné J, *et al. BMJ Immuno* 2026; 1:e000012. doi: 10.1136/bmjimm-2025-000012

Supplemental table 3: General demographic overview of each cohort

Demographics	Asthma (N=50)	Healthy Control (N=151)	P value
**Age (years) median (IQR)***	22 (20-26)	20 (19-22)	0.0005*
**Female – no. (%)**	38 (76)	82 (54)	0.007†
**Inoculum dose median (IQR) – CFU/nostril**	83,417 CFU/mL (78,333-85,292)	78,834 CFU/mL (76,333-84,167)	0.001*

*Mann Whitney U Test, †Pearson chi square

Supplemental table 4: Demographics and clinical characteristics of the asthma cohort by BTS treatment

	Full Cohort	BTS Step 2	BTS Step 3	P value
**Total participants n (%)**	50	36 (72)	14 (28)	NA
**Age (years) median (IQR)***	22 (19.0-26.0)	21 (19.0-25.0)	22 (19.0-27.0)	0.632
**Gender Female n (%) †**	38 (76)	28 (77.7)	10 (71.4)	0.637
**BMI (kg/m2) Mean (95 CI, SD) ‡**	24.6 (21.6-27.5)	25.3 (23.1 - 27.6, 6.7)	24.9(22.9 - 26.8, 3.3)	0.821
**Blood eosinophils Median (IQR)***	0.2 (0.1-0.4)	0.2 (0.1-0.4)	0.3 (0.1-0.4)	0.596
**FEV1% predicted Mean (95 CI, SD) ‡**	95 (91-100)	95 (91-100, 13.48)	94 (86-101,13.19)	0.752
**FeNO-ppb baseline Median (IQR)***	25.5 (13.8-49.2)	27 (12.0-50.5)	20.5 (14.8-49.2)	0.936
**PEFR variability% Median (IQR)***	14.6 (9.6-21.1)	14.3 (9.5-21.9)	14.8 (10.0-26.7)	0.756
**ACT score baseline Median (IQR)***	22 (20-24)	22 (20-24)	22(20-23)	0.278
**Inhaled Corticosteroid (μg) median (IQR)***	400 (400-400)	400 (400-400)	450 (400-800)	0.001
**Colonisation status n (%) †**	28 (56%)	19 (53%)	9 (64%)	0.462

BTS Treatment Steps are part of the British Thoracic Society (BTS) guidelines on management of asthma, where treatment is increased in stepwise manner -starting from short acting beta agonist at step 1, to oral steroids at step 5. These are described in online supplement Table
*Mann Whitney U Test, †Pearson chi square, ‡Independent Samples T test

Abbreviations: IQR (Inter quartile range); BMI (Body Mass Index); FEV (Forced expiratory volume); FeNO (Fractional Exhaled Nitric Oxide); PEFR (Peak expiratory flow rate) and ACT (Asthma Control Test).

Supplemental table 5: Demographics and clinical characteristics of the asthma cohort by colonisation status

	Full Cohort	Colonised	Non-Colonised	P value
**Number of Participants**	50	28 (56)	22 (44)	NA
**Age (years) median (IQR)***	22 (19.8-26.0)	22 (20-26)	21(19-24)	0.432
**Gender Female n (%)†**	38 (76)	23(82)	15(68)	0.251
**BMI (kg/m2) Median (IQR)‡**	24.6 (21.6-27.5)	24.7 (24.1-29.0)	23.5 (20.1-26.4)	0.019
**Blood eosinophils Median (IQR)***	0.2 (0.1-0.4)	0.2 (0.1-0.45)	0.25 (0.1-0.4)	0.933
**§Blood Eosinophils >0.3 n (%)†**	NA	16 (59%)	11 (41%)	0.615
**FEV1% predicted Median (IQR)‡**	95.5 (87.5-105)	98.5 (88.25-105)	94.5 (84.5-103.5)	0.340
**PEFR variability >12%‡**	NA	15 (62.5%)	13 (72%)	0.508
**FeNO-ppb baseline Median (IQR)***	25.5 (13.75-49.25)	20.5 (13.5-37.5)	29.5 (13.5-54.75)	0.287
**FeNO ppb Day 7 median (IQR)***	25 (11-41.5)	25 (10-37)	26.5 (13.75- 54.75)	0.526
**ᶫᶫFeNO >40 ppb n** (%)†**	NA	6 (37.5%)	10 (62.5%)	0.071
**ACT score baseline Median (IQR)‡**	22 (20.75-24)	22 (20.25-24)	22 (20.75-24)	0.129
**ACT score Day 29 median (IQR)‡**	22 (21-24)	22 (22-24)	22.5 (22-24)	0.816
**Inhaled Corticosteroid (µg) median (IQR)***	400 (400-400)	400 (400-500)	400 (350-400)	0.208

* Mann Whitney U Test, † Pearson chi square, ‡ Independent Samples T test, $ missing values. **n = number of participants with a raised FeNO in each cohort. ᶫᶫ Pearson's chi square p=0.071 for a raised FeNO of >40 at baseline for colonisation and for change in FeNO from baseline * Pearson's chi square p=0.367. §Pearson's Chi square p=0.615 for a raised eosinophil count of >0.3 at baseline, Pearson's chi square test p=0.274 for colonisation positive with steroid dose >400µg, and for steroid dose >500µg p=0.288

Supplemental table 6: Comparative analysis of the colonisation rates at different time points and duration between people with asthma compared to healthy individuals

	Asthma (N=50)	Healthy Controls (N=151)	P value
**Any timepoint– no (%)**	28 (56)	68 (45)	0.178⁺
**day 2 (%)**	19 (38)	61 (40)	0.764⁺
**day 7 (%)**	22 (44)	62(41)	0.715⁺
**day 9 (%)**	17 (34)	60 (40)	0.470⁺
**day 14 (%)**	19 (38)	50 (33)	0.528⁺
**day 22 (%)**	12 (24)	43 (28)	0.538⁺
**day 29 (%)**	11 (22)	34 (23)	0.939⁺
**Duration days median (range)**	14 (7-29)	29 (14-29)	0.034*
**Colonisation Density Log transformed AUC (CFU/mL) median (range)**	63.49 (14.04-116.3)	81.18 (48.15-104.5)	0.060*

[†]Pearson chi square, [*]Mann Whitney U Test

Supplemental table 7: Statistical summary of baseline cytokine comparisons from figure 5A.

Figure	Cytokine	Asthma vs Healthy	Statistical test	Correction test	p value	Significant (Y/N)
5A	EGF	No difference	Paired t-test (log2 fold-change)	Benjamini–Hochberg (p < 0.01)	p<0.01	No
5A	Eotaxin	No difference	Paired t-test (log2 fold-change)	Benjamini–Hochberg (p < 0.01)	p<0.01	No
5A	FGF-basic	Lower in asthma	Paired t-test (log2 fold-change)	Benjamini–Hochberg (p < 0.01)	p<0.01	Yes
5A	G-CSF	Lower in asthma	Paired t-test (log2 fold-change)	Benjamini–Hochberg (p < 0.01)	p<0.01	Yes
5A	GM-CSF	Lower in asthma	Paired t-test (log2 fold-change)	Benjamini–Hochberg (p < 0.01)	p<0.01	Yes
5A	HGF	Lower in asthma	Paired t-test (log2 fold-change)	Benjamini–Hochberg (p < 0.01)	p<0.01	Yes
5A	IFN-alpha	Lower in asthma	Paired t-test (log2 fold-change)	Benjamini–Hochberg (p < 0.01)	p<0.01	Yes
5A	IFN-gamma	Lower in asthma	Paired t-test (log2 fold-change)	Benjamini–Hochberg (p < 0.01)	p<0.01	Yes
5A	IL-10	Lower in asthma	Paired t-test (log2 fold-change)	Benjamini–Hochberg (p < 0.01)	p<0.01	Yes
5A	IL-12	Lower in asthma	Paired t-test (log2 fold-change)	Benjamini–Hochberg (p < 0.01)	p<0.01	Yes
5A	IL-13	Lower in asthma	Paired t-test (log2 fold-change)	Benjamini–Hochberg (p < 0.01)	p<0.01	Yes
5A	IL-15	Lower in asthma	Paired t-test (log2 fold-change)	Benjamini–Hochberg (p < 0.01)	p<0.01	Yes
5A	IL-17A	No difference	Paired t-test (log2 fold-change)	Benjamini–Hochberg (p < 0.01)	p<0.01	No
5A	IL-1b	Lower in asthma	Paired t-test (log2 fold-change)	Benjamini–Hochberg (p < 0.01)	p<0.01	Yes
5A	IL-1RA	Higher in asthma	Paired t-test (log2 fold-change)	Benjamini–Hochberg (p < 0.01)	p<0.01	Yes
5A	IL-2	Lower in asthma	Paired t-test (log2 fold-change)	Benjamini–Hochberg (p < 0.01)	p<0.01	Yes
5A	IL-2R	Lower in asthma	Paired t-test (log2 fold-change)	Benjamini–Hochberg (p < 0.01)	p<0.01	Yes
5A	IL-4	No difference	Paired t-test (log2 fold-change)	Benjamini–Hochberg (p < 0.01)	p<0.01	No
5A	IL-5	No difference	Paired t-test (log2 fold-change)	Benjamini–Hochberg (p < 0.01)	p<0.01	No
5A	IL-6	Lower in asthma	Paired t-test (log2 fold-change)	Benjamini–Hochberg (p < 0.01)	p<0.01	Yes
5A	IL-7	Lower in asthma	Paired t-test (log2 fold-change)	Benjamini–Hochberg (p < 0.01)	p<0.01	Yes

5A	IL-8	Lower in asthma	Paired t-test (log2 fold-change)	Benjamini–Hochberg ($p < 0.01$)	p<0.01	Yes
5A	IP-10	Lower in asthma	Paired t-test (log2 fold-change)	Benjamini–Hochberg ($p < 0.01$)	p<0.01	Yes
5A	MCP-1	Lower in asthma	Paired t-test (log2 fold-change)	Benjamini–Hochberg ($p < 0.01$)	p<0.01	Yes
5A	MIG	No difference	Paired t-test (log2 fold-change)	Benjamini–Hochberg ($p < 0.01$)	p<0.01	No
5A	MIP-1alpha	Lower in asthma	Paired t-test (log2 fold-change)	Benjamini–Hochberg ($p < 0.01$)	p<0.01	Yes
5A	MIP-1beta	Lower in asthma	Paired t-test (log2 fold-change)	Benjamini–Hochberg ($p < 0.01$)	p<0.01	Yes
5A	RANTES	No difference	Paired t-test (log2 fold-change)	Benjamini–Hochberg ($p < 0.01$)	p<0.01	No
5A	TNF-alpha	Lower in asthma	Paired t-test (log2 fold-change)	Benjamini–Hochberg ($p < 0.01$)	p<0.01	Yes
5A	VEGF	Lower in asthma	Paired t-test (log2 fold-change)	Benjamini–Hochberg ($p < 0.01$)	p<0.01	Yes

Supplemental table 7. Statistical summary of baseline cytokine comparisons. Baseline nasal lining fluid cytokine concentrations were log-transformed and compared between individuals with asthma and healthy controls using two-sample t-tests. P values were adjusted for multiple testing across the 30 cytokines using the Benjamini–Hochberg method. Twenty-two cytokines were significantly lower, and one cytokine (IL-1RA) was significantly higher in individuals with asthma after correction; the remaining cytokines showed no statistically significant differences.

Supplemental table 8: Statistical summary of nasal cytokines responses following colonisation from figures 5B and C.

Figure	Group	Colonisation status	Time points vs. Baseline	Statistical test	Correction Test	Significant (Y/N)
5B	Asthma	Colonised (C+)	Day 2 vs. Baseline	Paired t-test (log2 fold-change)	Benjamini–Hochberg (p < 0.01)	No
5B	Asthma	Colonised (C+)	Day 7 vs. Baseline	Paired t-test (log2 fold-change)	Benjamini–Hochberg (p < 0.01)	No
5B	Asthma	Colonised (C+)	Day 9 vs. Baseline	Paired t-test (log2 fold-change)	Benjamini–Hochberg (p < 0.01)	No
5B	Asthma	Colonised (C+)	Day 29 vs. Baseline	Paired t-test (log2 fold-change)	Benjamini–Hochberg (p < 0.01)	No
5B	Asthma	Non-colonised (C-)	Day 2 vs. Baseline	Paired t-test (log2 fold-change)	Benjamini–Hochberg (p < 0.01)	No
5B	Asthma	Non-colonised (C-)	Day 7 vs. Baseline	Paired t-test (log2 fold-change)	Benjamini–Hochberg (p < 0.01)	No
5B	Asthma	Non-colonised (C-)	Day 9 vs. Baseline	Paired t-test (log2 fold-change)	Benjamini–Hochberg (p < 0.01)	No
5B	Asthma	Non-colonised (C-)	Day 29 vs. Baseline	Paired t-test (log2 fold-change)	Benjamini–Hochberg (p < 0.01)	No
5C	Healthy controls	Colonised (C+)	Day 2 vs. Baseline	Paired t-test (log2 fold-change)	Benjamini–Hochberg (p < 0.01)	No
5C	Healthy controls	Colonised (C+)	Day 7 vs. Baseline	Paired t-test (log2 fold-change)	Benjamini–Hochberg (p < 0.01)	No
5C	Healthy controls	Colonised (C+)	Day 9 vs. Baseline	Paired t-test (log2 fold-change)	Benjamini–Hochberg (p < 0.01)	No
5C	Healthy controls	Colonised (C+)	Day 29 vs. Baseline	Paired t-test (log2 fold-change)	Benjamini–Hochberg (p < 0.01)	No
5C	Healthy controls	Non-colonised (C-)	Day 2 vs. Baseline	Paired t-test (log2 fold-change)	Benjamini–Hochberg (p < 0.01)	No
5C	Healthy controls	Non-colonised (C-)	Day 7 vs. Baseline	Paired t-test (log2 fold-change)	Benjamini–Hochberg (p < 0.01)	No
5C	Healthy controls	Non-colonised (C-)	Day 9 vs. Baseline	Paired t-test (log2 fold-change)	Benjamini–Hochberg (p < 0.01)	No
5C	Healthy controls	Non-colonised (C-)	Day 29 vs. Baseline	Paired t-test (log2 fold-change)	Benjamini–Hochberg (p < 0.01)	No

Supplemental table 8. Statistical summary of the longitudinal nasal cytokine responses following pneumococcal inoculation (Figures 5B and C). Paired t-tests on $\log_2$ fold-change values were used to compare cytokine levels at each post-inoculation time point with baseline within each cohort and stratified by colonisation status. P values were adjusted for multiple testing using the Benjamini–Hochberg method. No statistically significant differences were observed at any time point after correction.

Reiné J, *et al. BMJ Immuno* 2026; 1:e000012. doi: 10.1136/bmjimm-2025-000012

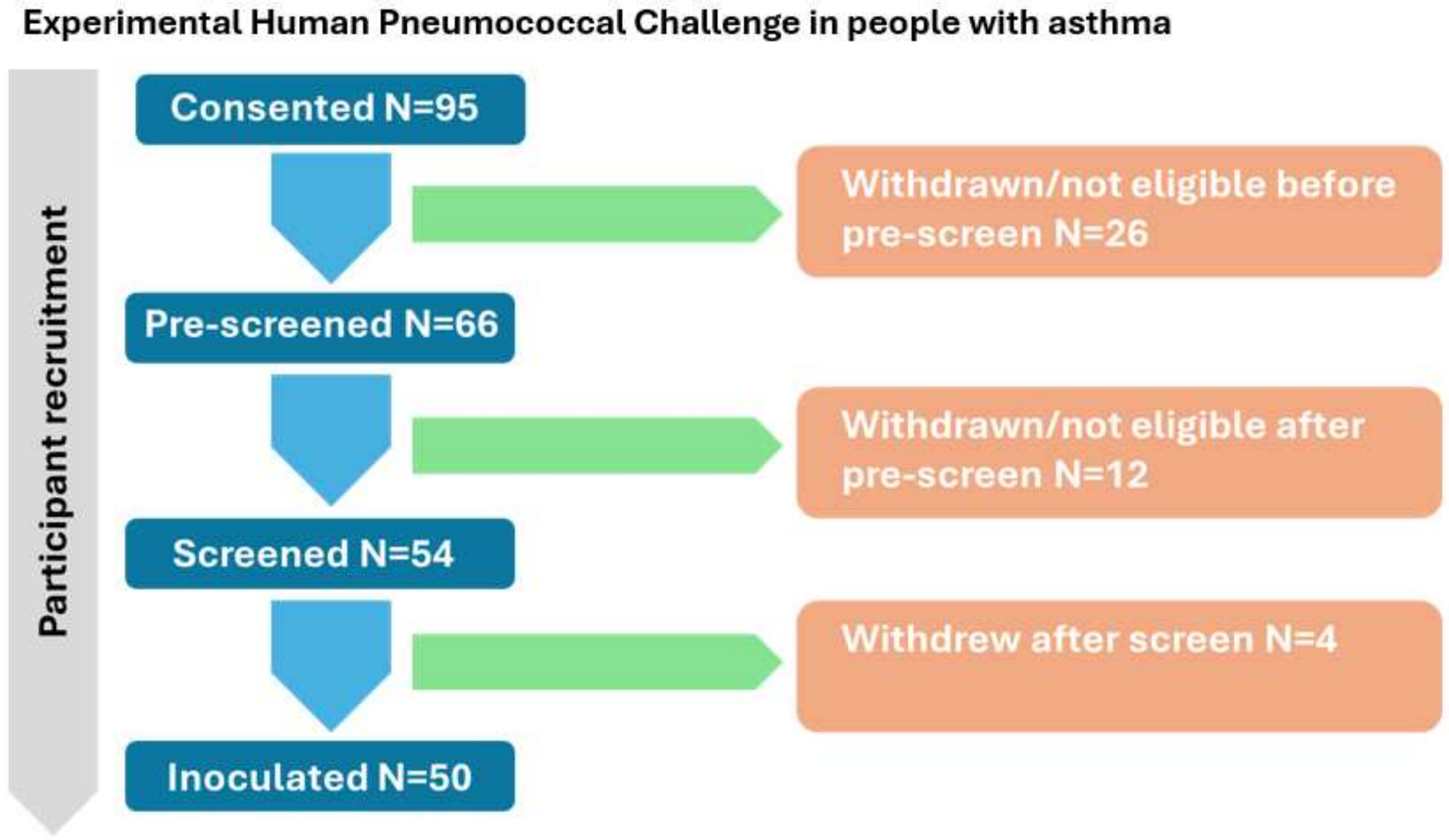

Supplemental Figure 1: Recruitment and retention. This flow chart outlines participant recruitment and retention. Reasons for withdrawal include: not using inhaled corticosteroids (ICS) (n=17), inability to contact after consent (n=5), medical placement (n=2), excessive ICS dosage (n=2), and a variety of individual factors (n=1 each): non-compliance with ICS use, relocation, unspecified reasons, personal issues, elevated white blood cell count, daily headaches, penicillin allergy, bronchiectasis, use of non-invasive ventilation, consent withdrawal, >3 exacerbations in the past 12 months, and diagnosed pneumococcal pneumonia. One participant was excluded after day 2 due to a low peak expiratory flow rate (PEFR) and the need for increased ICS, surpassing the 800µg budesonide equivalent threshold.

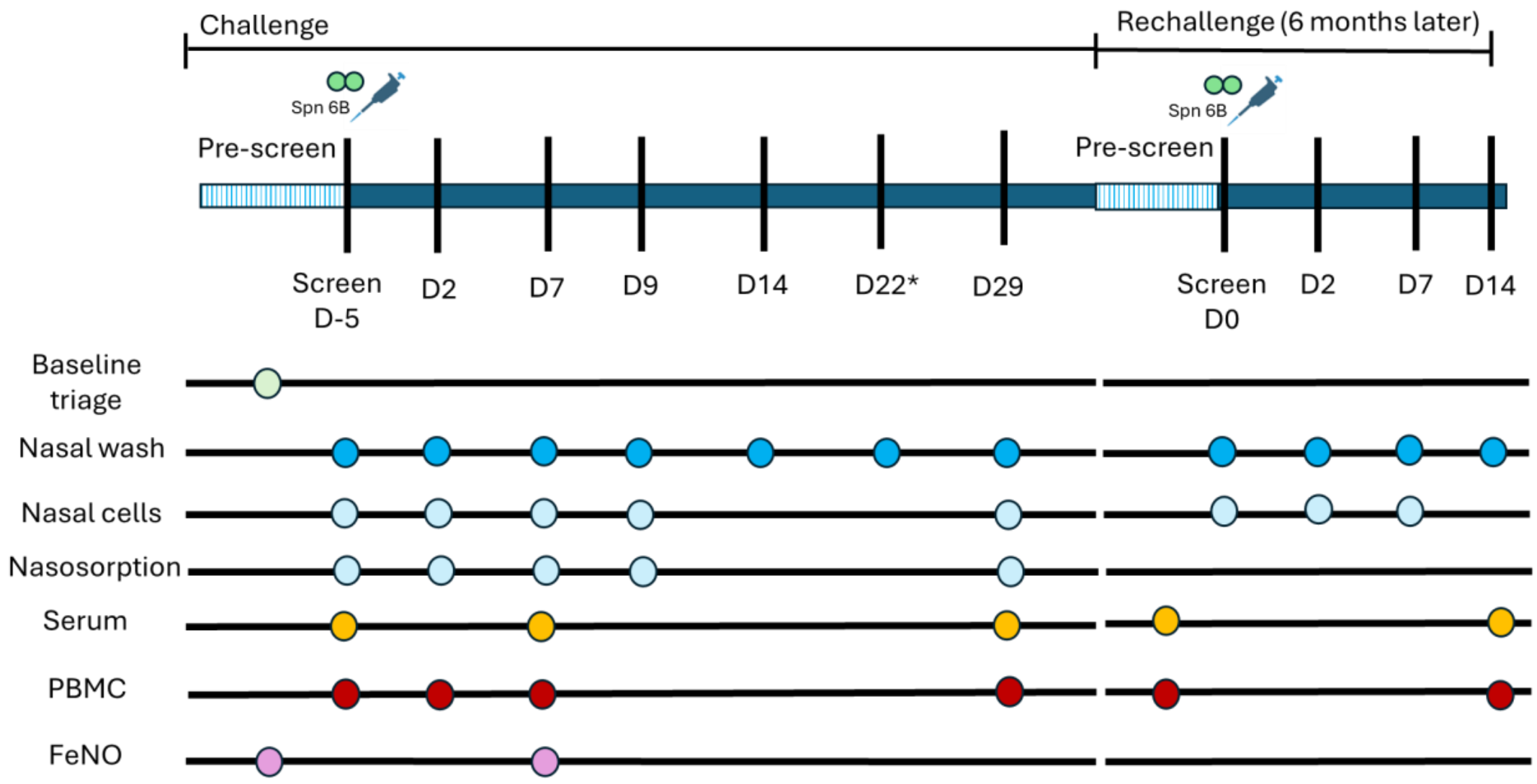

Supplemental Figure 2: EHPC Asthma Study Overview and Sampling Schedule. This diagram illustrates the study design and follow-up visit timepoints. Participants were pre-screened up to 14 days before the baseline visit, where a comprehensive history and examination were performed, along with spirometry, Peak Expiratory Flow Rate (PEFR), and Fractional Exhaled Nitric Oxide (FeNO) testing. Nasal washes samples were collected for pneumococcal detection, while serum and nasal lining fluid (nasosorption or NSP) samples were collected for MSD/Luminex analysis. Nasal cells and PBMCs were obtained for cellular immunophenotyping. Daily contact during the first week included recording of peak flow and temperature. Participants maintained a peak flow diary and symptom log and were instructed to follow their personal asthma action plan. *Only colonized participants included for FeNO testing.

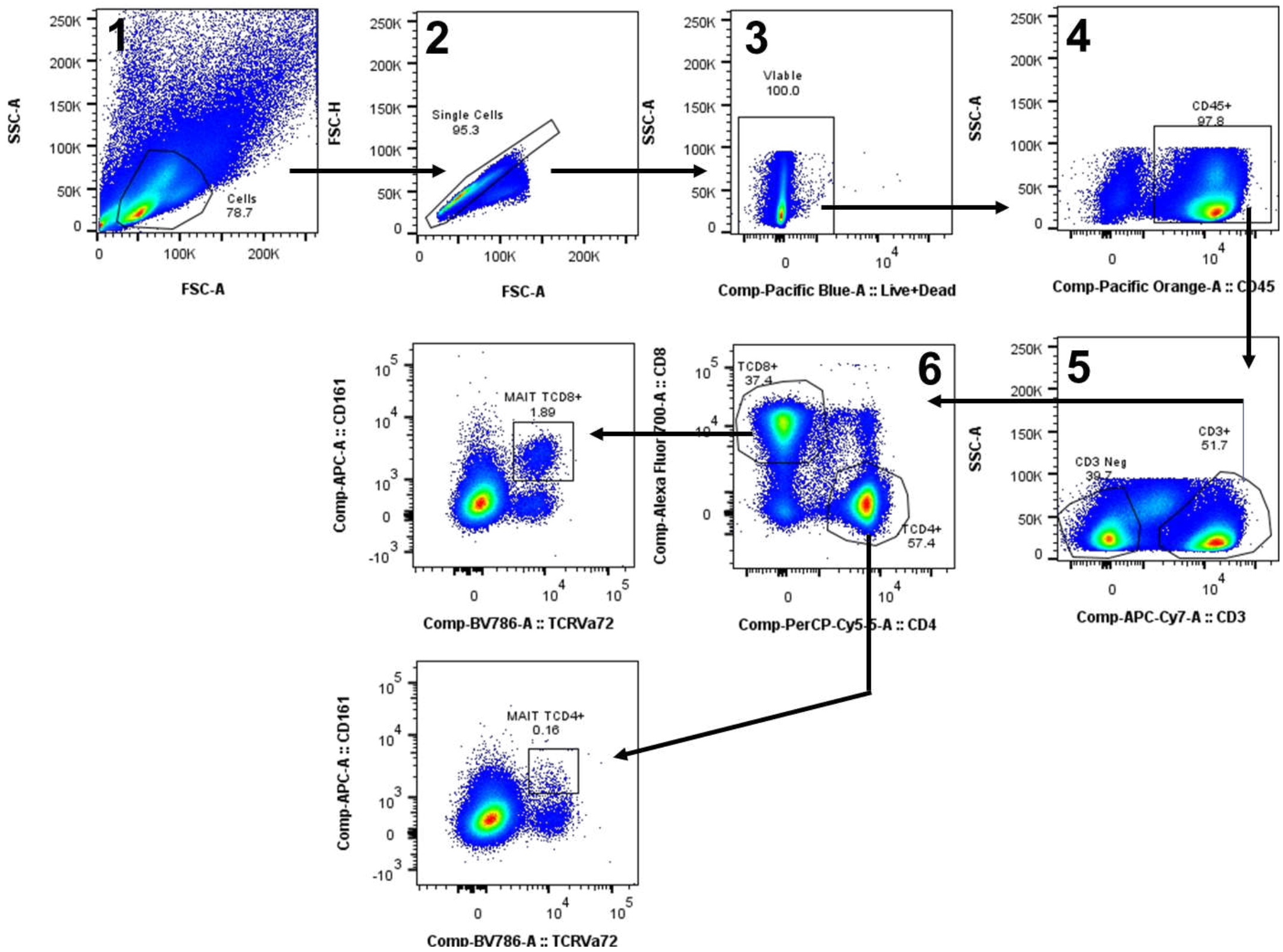

Supplemental Figure 3: Mucosal-associated invariant T (MAIT) cell immunophenotyping in peripheral blood mononuclear cells (PBMC) from a representative asthma participant. 1) Identification of lymphocyte gate 2) Exclusion of doublets with the identification of singlets improving the accuracy of the analysis 3) Selection of the viable cells 4) Identification of immune cells based on CD45+ expression 5) Identification of T cells according to CD3+ expression 6) Characterization of the different T cellular subsets ($CD4^+$ and $CD8^+$ T cells) and subsequent identification of MAIT cells from each T cell subset.

Reiné J, *et al. BMJ Immuno* 2026; 1:e000012. doi: 10.1136/bmjimm-2025-000012

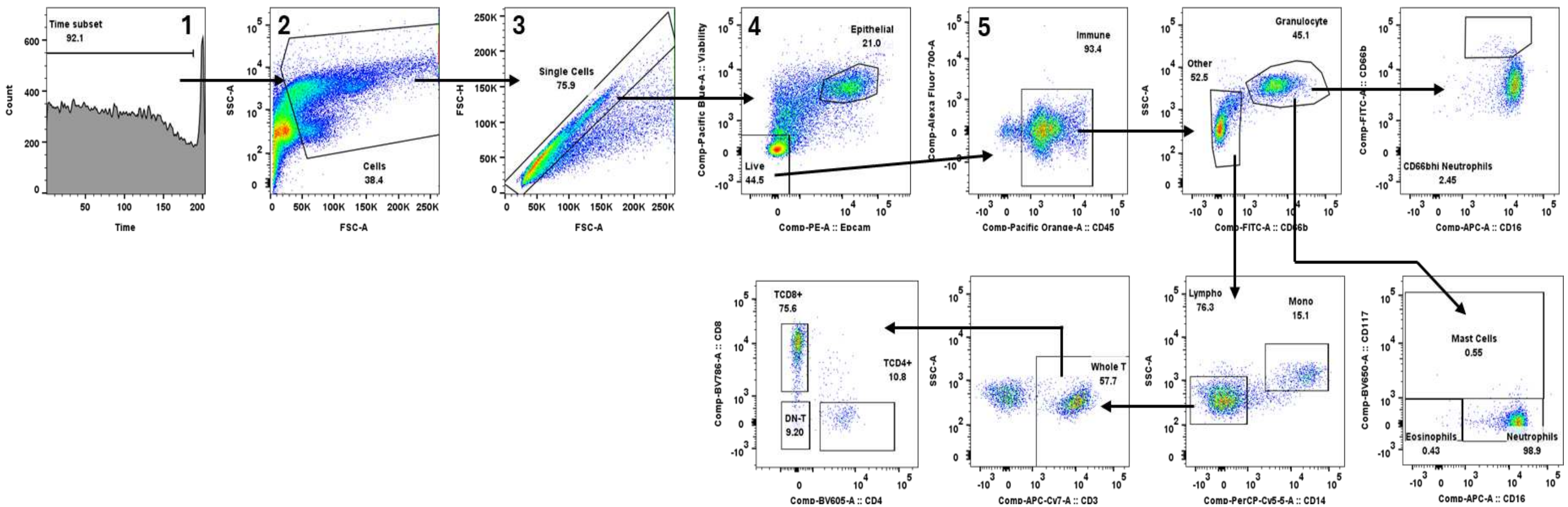

Supplemental Figure 4: Nasal cell immunophenotyping. Flow cytometry gating strategy of the major cell subsets from a representative asthma participant. Identification of the different nasal immunological cellular subsets according to 1) adequate identification of cells acquired with a stable acquisition over time 2) Identification of nasal cells as previously described 3) exclusion of doublets with the identification of singlets improving the accuracy of the analysis 4) Selection of the viable cells and epithelial cells 5) Identification of immune cells based on CD45+ expression, and subsequent analysis of different subsets of granulocytes, monocytes, T cells and their cellular subsets.

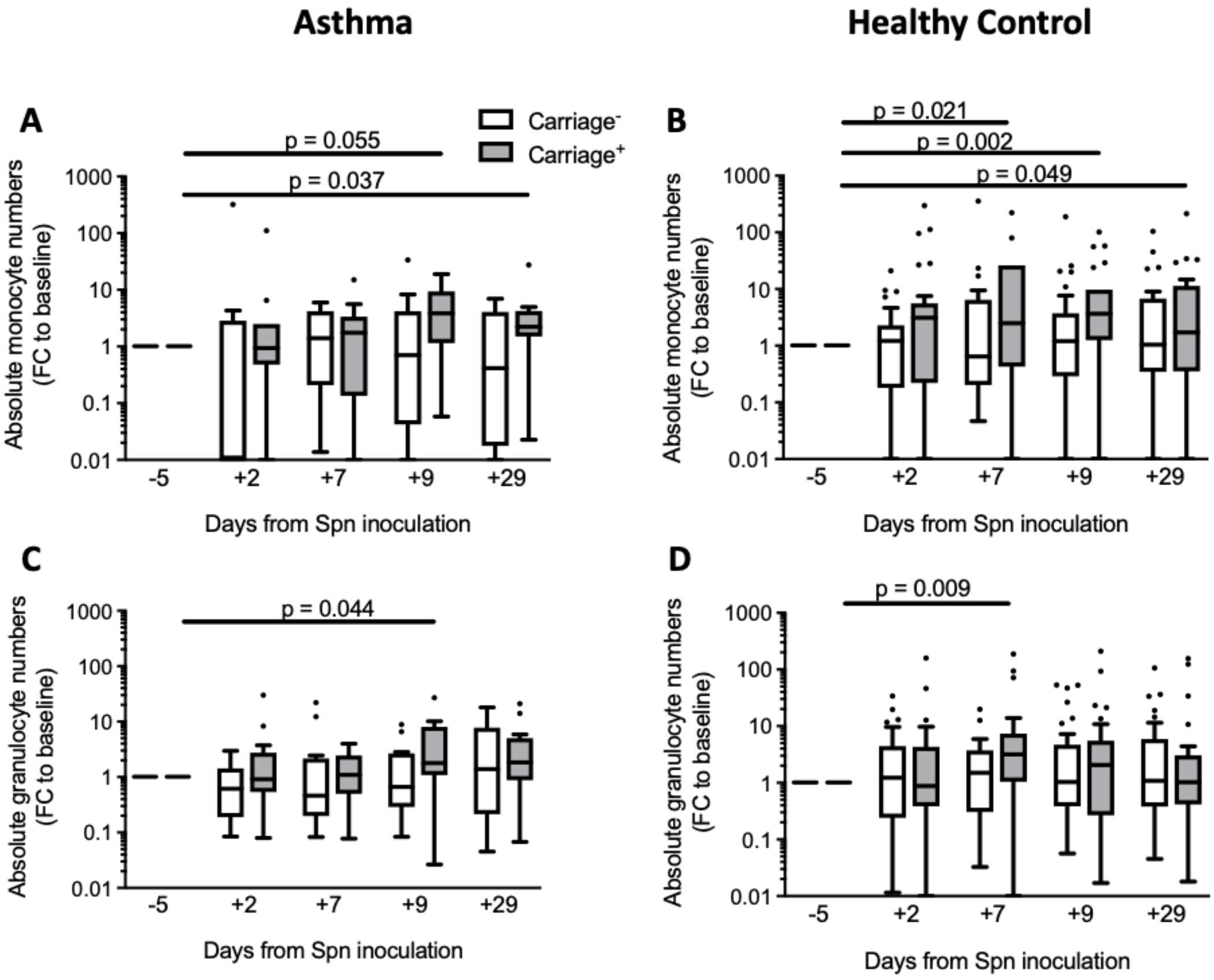

Supplemental Figure 5: Changes in baseline-normalised nasal monocyte and granulocyte counts in asthma and healthy individuals. Baseline-normalized monocytes count in A) asthma and B) healthy individuals, and baseline-normalized granulocytes counts in C) asthma and D) healthy individuals. Turkey boxplots are presented, along with results from paired-Wilcoxon rank comparing changes from baseline to days 2, 7, 9, and 29. Carriage positive is indicated with grey boxplots and carriage negative is indicated with white boxplots in each case. [FC= Fold Change; Spn = *S. pneumoniae*.]

Reiné J, *et al. BMJ Immuno* 2026; 1:e000012. doi: 10.1136/bmjimm-2025-000012

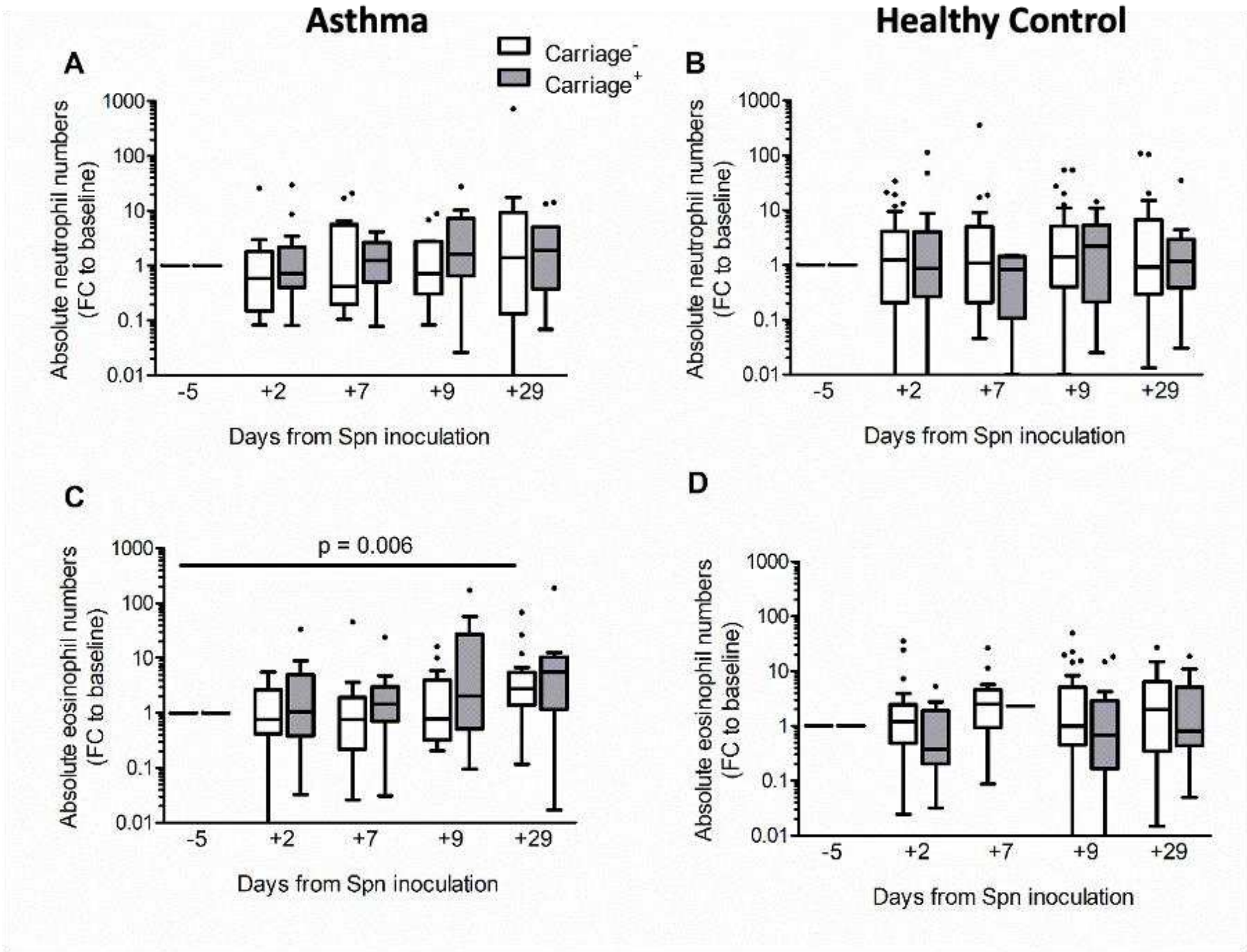

Supplemental Figure 6: Changes in baseline-normalised nasal neutrophil and eosinophil counts in asthma and healthy individuals. Baseline-normalized neutrophils count in A) asthma and B) healthy individuals. Baseline-normalized eosinophils count in C) asthma and D) healthy individuals. Turkey boxplots are presented, along with the results from paired-Wilcoxon rank tests comparing changes from baseline to the days 2, 7, 9, and 29. Carriage positive is indicated with grey boxplots and carriage negative is indicated with white boxplots in each case. [FC= Fold Change; Spn = *S. pneumoniae*.]

Reiné J, *et al. BMJ Immuno* 2026; 1:e000012. doi: 10.1136/bmjimm-2025-000012

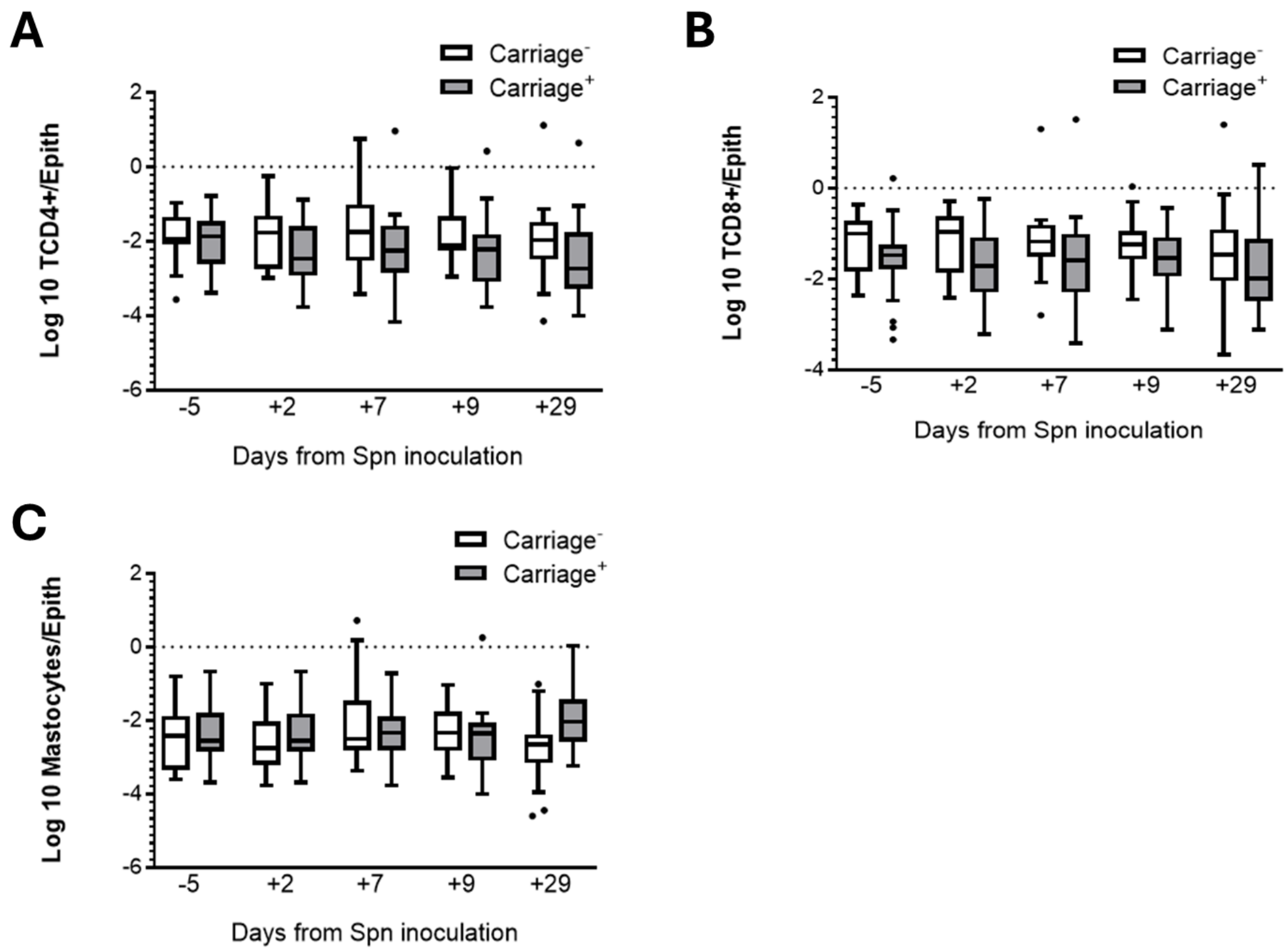

Supplemental Figure 7: Longitudinal changes of classical CD4⁺ and CD8⁺ T cell frequencies, and mast cells counts normalised to epithelial cells from baseline (D-5) and following colonisation (days 2, 7, 9, and 29) in people with asthma. A and B) Normalized CD4⁺T and CD8⁺T cell counts following colonisation in people in asthma. No significant differences were observed within classical T cells following colonisation C) Normalized mast cells counts following colonisation in people with asthma. No differences were observed within mast cells following colonisation. Turkey boxplots are presented, along with the results from paired-Wilcoxon rank tests comparing changes from baseline to the days 2, 7, 9, and 29. Carriage positive is indicated with grey boxplots, and carriage negative is indicated with white boxplots in each case. [FC= Fold Change; Spn = *S. pneumoniae*.]

Reiné J, *et al. BMJ Immuno* 2026; 1:e000012. doi: 10.1136/bmjimm-2025-000012

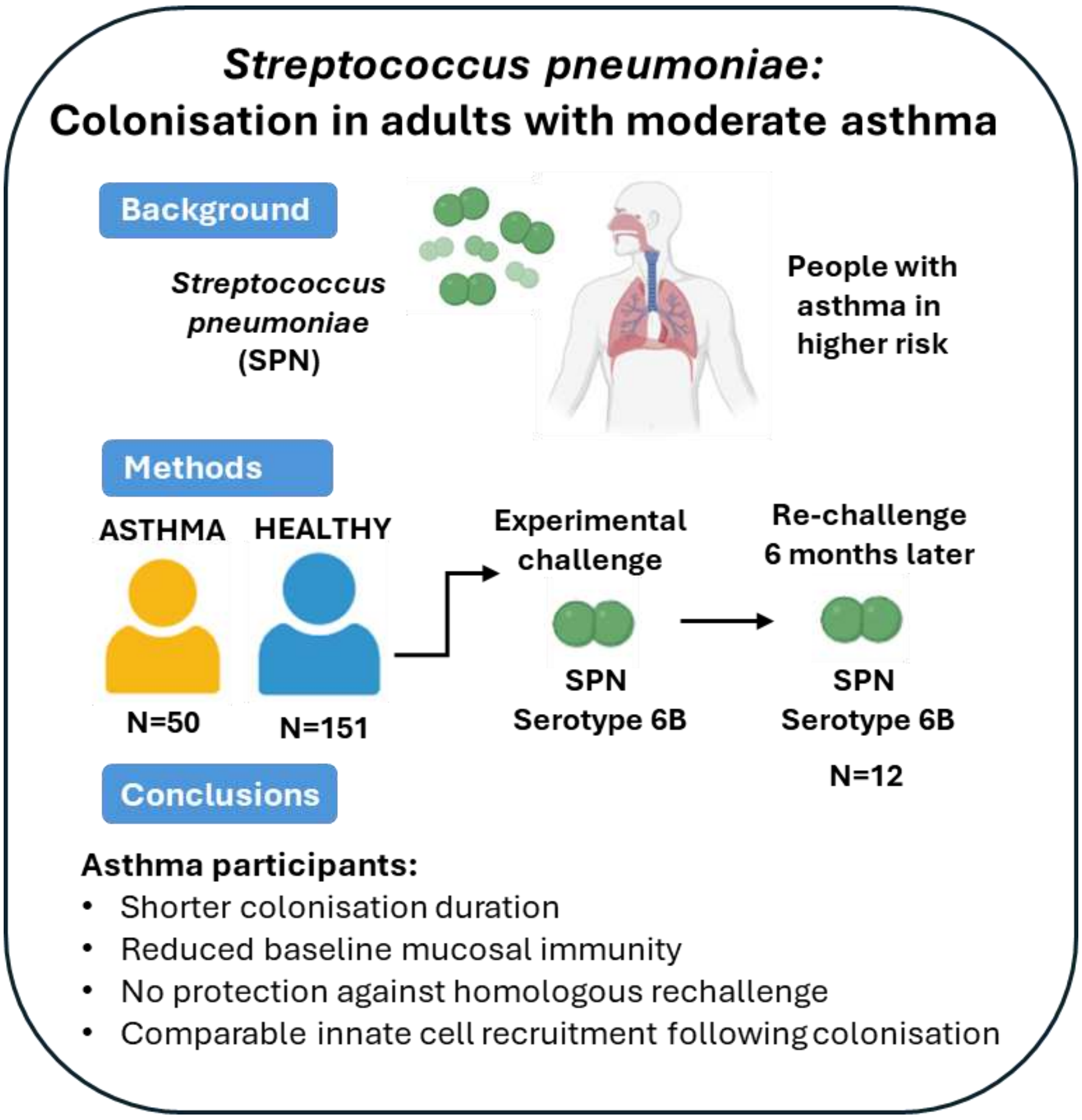

Supplemental figure 8: Graphical abstract summarising an experimental human controlled pneumococcal challenge comparing pneumococcal colonisation dynamics in adults with asthma and healthy participants. Asthma participants showed shorter colonisation duration, reduced baseline mucosal immunity, and no protection against homologous rechallenge, despite comparable innate cell recruitment following colonisation. Figure created with Biorender.

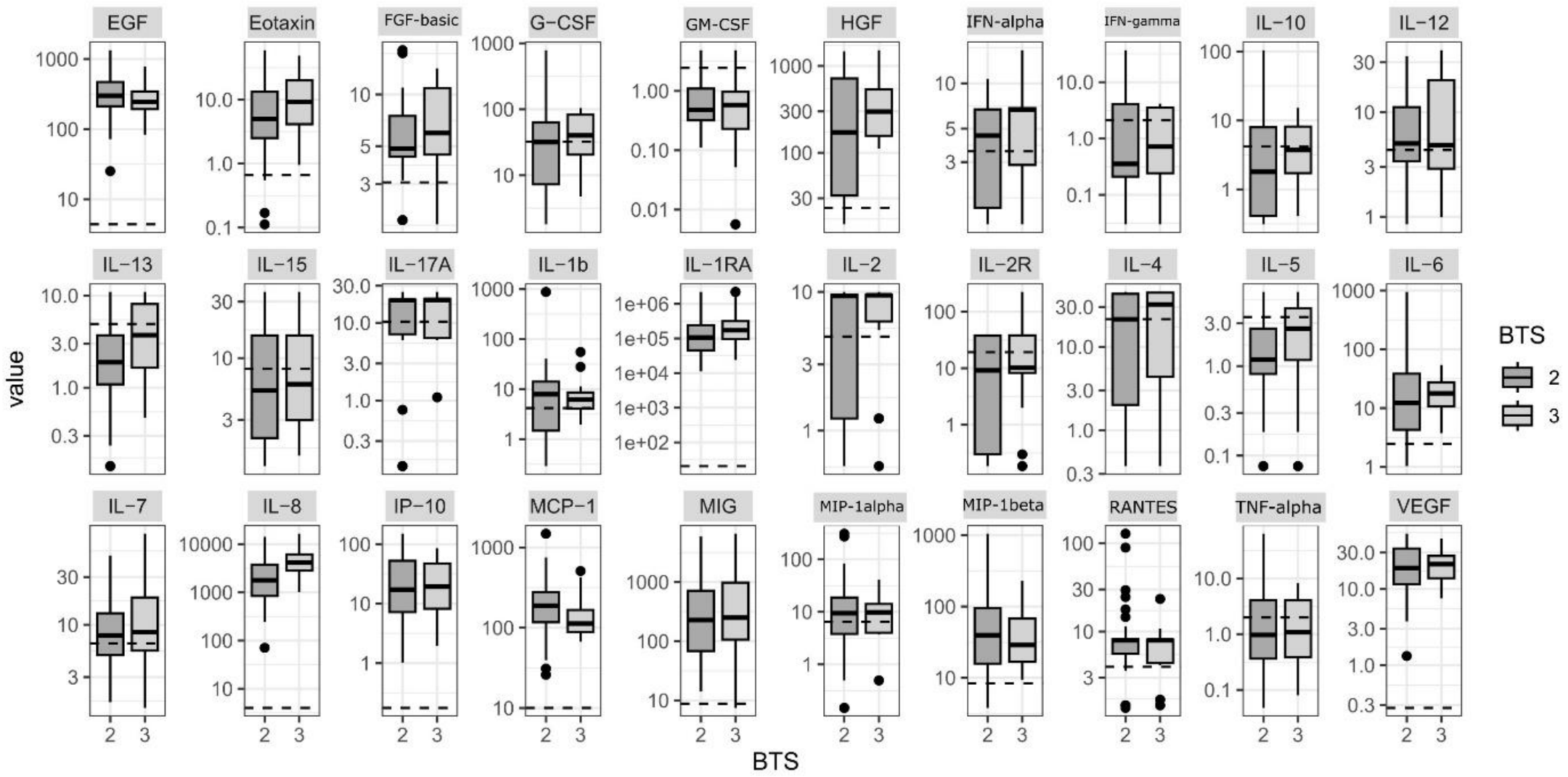

Supplemental figure 9: Comparative nasal cytokine profiles in asthma participants stratified according to their British Thoracic Society (BTS) treatment step. Asthma participants were stratified according to BTS treatment step (Step 2: low-dose inhaled corticosteroids; Step 3: combination therapy or higher-dose inhaled corticosteroids). Cytokine levels were measured from nasal lining fluid (nasosorption) and compared between both asthma groups. Boxplots represent data for each group (BTS 2 vs. BTS 3), with dashed horizontal lines indicating the average value of the lowest standard across all plates for each cytokine. No statistically significant differences were observed in nasal cytokine profiles across BTS treatment steps within the asthma cohort, after correction for multiple testing.

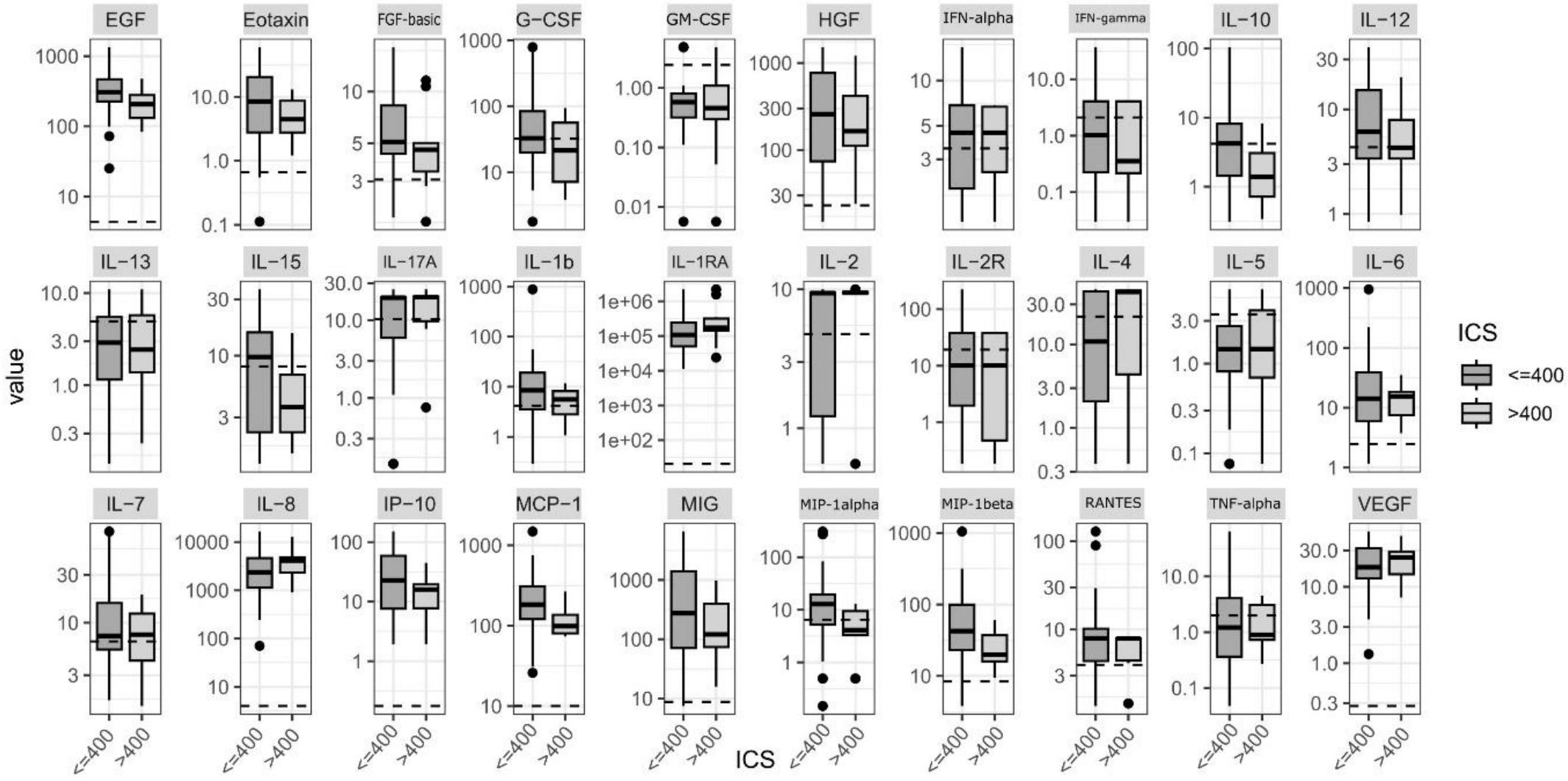

Supplemental figure 10: Comparative nasal cytokine profiles in asthma participants according to inhaled corticosteroid (ICS) dose (≤400 µg and >400 µg). Cytokine concentrations were measured from nasal lining fluid (nasosorption) and compared between both groups to assess the potential influence of corticosteroid dose on mucosal immune profiles. Boxplots represent data for each group (ICS dosage: (≤400 µg and >400 µg), with dashed horizontal lines indicating the average value of the lowest standard across all plates for each cytokine. No statistically significant differences were observed in nasal cytokine profiles across ICS dosage within the asthma cohort, after correction for multiple testing.

Reiné J, *et al. BMJ Immuno* 2026; 1:e000012. doi: 10.1136/bmjimm-2025-000012

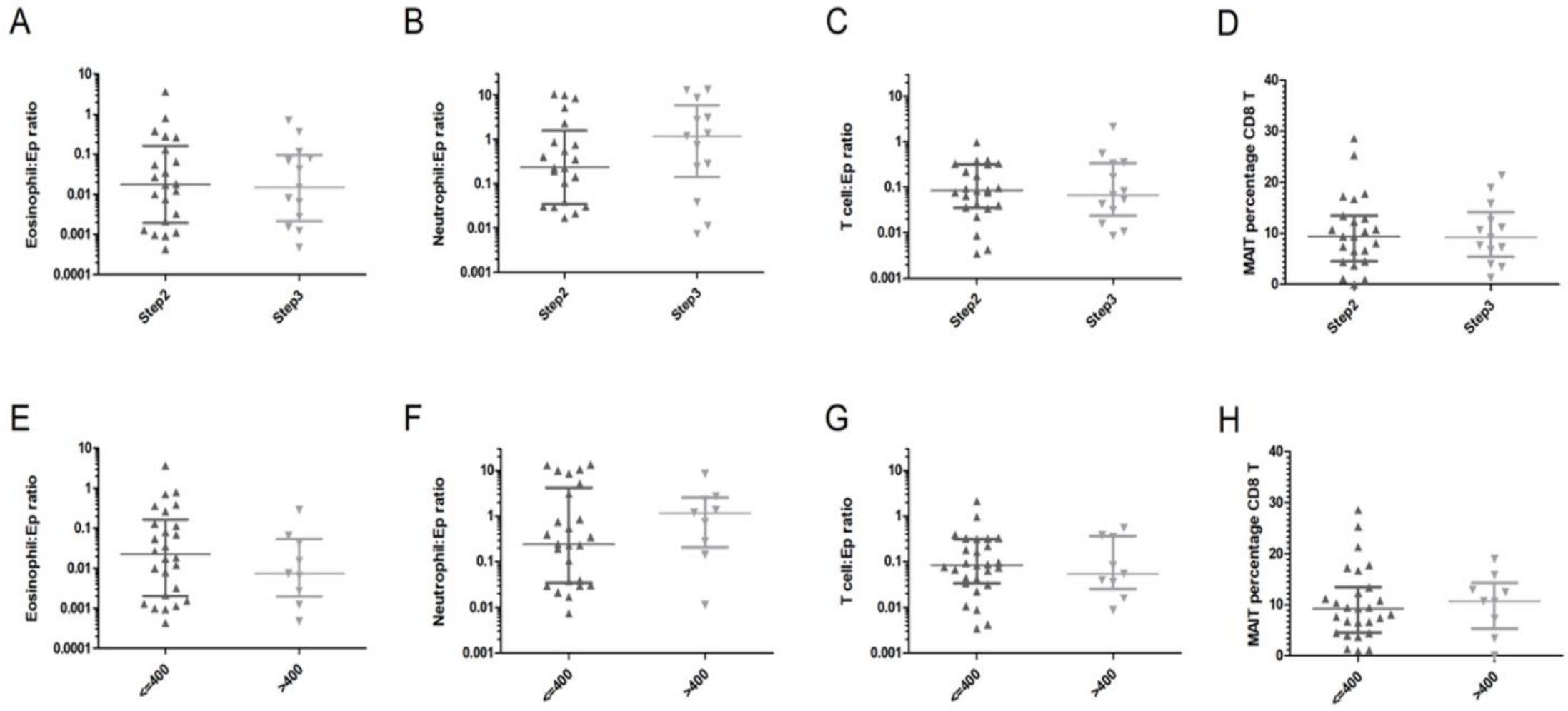

Supplemental figure 11: Nasal immune profiles in asthma participants stratified by British Thoracic Society (BTS) treatment step (Step 2 vs. Step 3, top row) and inhaled corticosteroid (ICS) dose (≤400 µg vs >400 µg, bottom row). (A-C) Nasal eosinophils, neutrophils, T cells levels normalised to epithelial cells in individuals with asthma stratified according to their BTS treatment. (D) Percentage of MAIT CD8+ T cells according to BTS treatment. (E-G) Nasal eosinophils, neutrophils, T cells levels normalised to epithelial cells in individuals with asthma stratified according to their ICS dosage. (H) Percentage of MAIT CD8+ T cells according to ICS dosage. Individual values are shown, as well as median and interquartile ranges.

Reiné J, *et al. BMJ Immuno* 2026; 1:e000012. doi: 10.1136/bmjimm-2025-000012

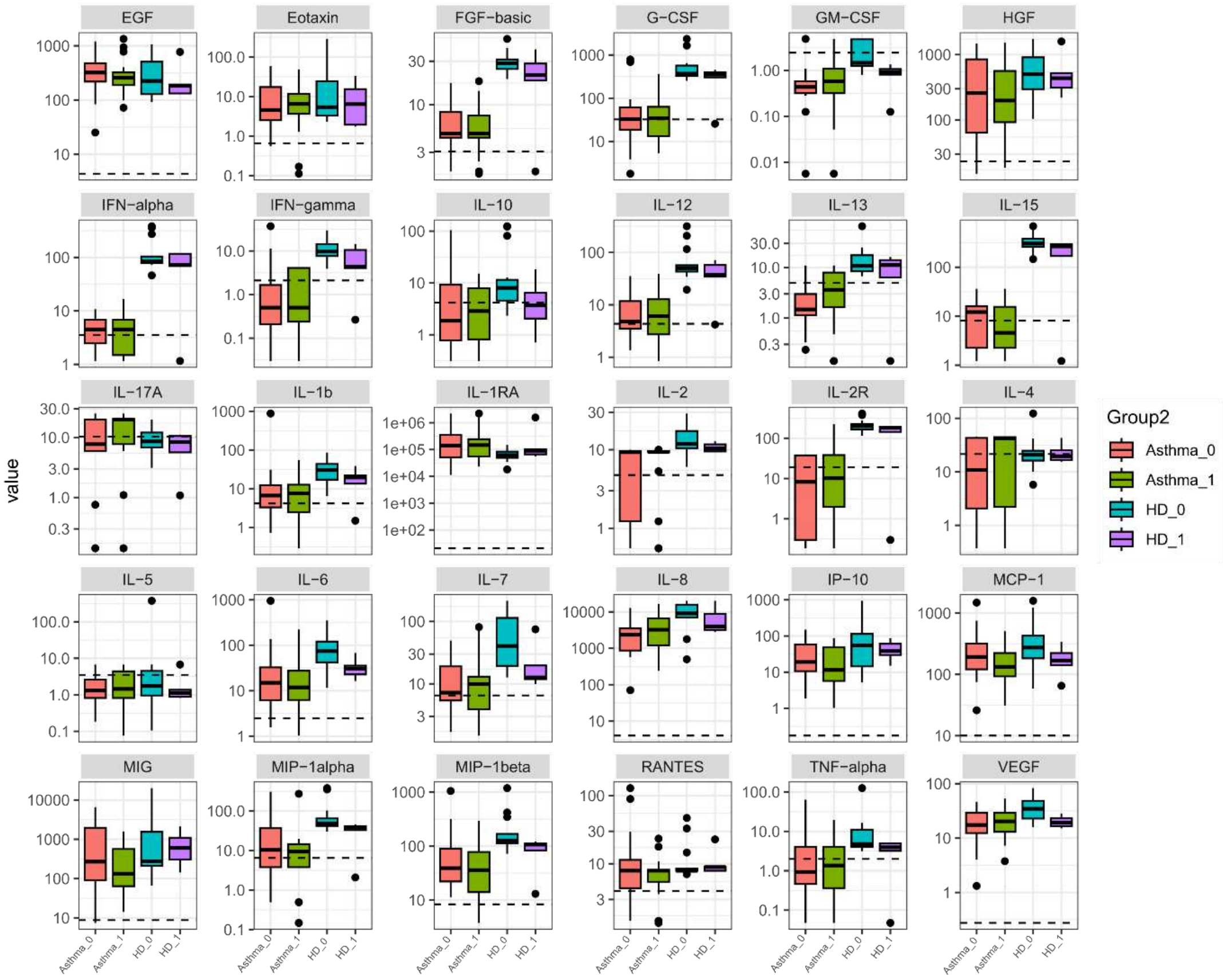

Supplemental Figure 12. Baseline nasal cytokine profiles stratified by colonisation status. Baseline nasal lining fluid cytokine concentrations in individuals with asthma and healthy controls, stratified by colonisation status (colonised and non-colonised). No statistically significant differences were observed within the asthma cohort between colonised and non-colonised participants, nor within the healthy control cohort between colonised and non-colonised participants, for any of the cytokines analysed. Boxplots represent data for each group: asthma colonised (red), asthma non-colonised (green), healthy controls colonised (blue), and healthy controls non-colonised (violet). Dashed horizontal lines indicate the mean value of the lowest standard across all plates for each cytokine.

Reiné J, *et al. BMJ Immuno* 2026; 1:e000012. doi: 10.1136/bmjimm-2025-000012

Supplemental Methods 1. Pneumococcal proteins used for meso-scale discovery analysis were: Spr2021, PcpA, StkP, RrgB231, PcsB, PiaA, PhtD, WtPly, Ply, Spr0057, PsaA, PspC, Spr1, LytC, PspAUAB099, NanA, PhtDD, PiuA, RygA, SP0609, Spr0096, PhtE, LytB, RrgB, SP2194, RrgBT4, PspAUAB055

Supplemental Methods 2. Core immunophenotyping cocktail included Epcam-PE, HLADR-PECy7, CD66b-FITC (all Biolegend), CD3-APC-H7 (BD Biosciences) or CD3-APCCy7 (Biolegend), CD14-PerCPCy5.5 (BD Biosciences) and CD45-PacOrange (Thermo Fisher Scientific). Additional phenotyping additionally used BDCA2-APC (Miltenyi Biotech) and/or CD16-APC, CD1c-PE/Dazzle594, CD19-BV650, CD4-BV605, CD8-BV785, TCRvα7.2-PE/Dazzle594, TCRvα7.2-BV711, CD123–BV711 and/or CD117–BV650 (all Biolegend).

Supplemental Methods 3. Cytokine and immune protein panel included: G-CSF, GM-CSF, IFN-α, IFN-γ, IL-1β, IL-1RA, IL-2, IL-2R, IL-4, IL-5, IL-6, IL-7, IL-8, IL-10, IL-12 (p40/p70), IL-13, IL-15, IL-17A, TNF-α, Eotaxin, IP-10, MCP-1, MIG, MIP-1α, MIP-1β, Rantes, EGF, FGF-β, HGF and VEGF. Preparation and analysis were performed according to manufacturer's instructions and used xPonent3.1 software.

Reiné J, *et al. BMJ Immuno* 2026; 1:e000012. doi: 10.1136/bmjimm-2025-000012
